# Supplementary material for: Primate tooth crown nomenclature revisited
Source: PeerJ. 2023 Jan 12;11:e14523. doi: 10.7717/peerj.14523 (PMC9840859; doi:10.7717/peerj.14523)
Supplement: File S1 [file peerj-11-14523-s001.docx]

**Supporting File**

**Accession numbers of all specimens studied**

**Lemuridae**

*Prolemur simus –* ZMB 5511

*Hapelemur griseus –* ZMB 43240, ZMB 7707

*Eulemur fulvus –* ZMB 44476, ZMB 7766

*Varecia variegate –* ZMB MAM_A, ZMB 12928

**Lepilemuridae**

*Lepilemur leucopus –* ZMB 35351, ZMB 44655

*Lepilemur mustelinus –* ZMB 5649

**Cheirogaleidae**

*Phaner furcifer* – ZMB 3838

*Microcebus sp. –* ZMB 44624, ZMB 44625

*Cheirogaleus sp. –* ZMB 4464, ZMB 4823, ZMB 35352,

**Indriidae**

*Propithecus diadema* – ZMB 44687

*Indri indri* – ZMB 4333, ZMB 84280

*Avahi laniger* – ZMB 4686, ZMB 44675

**Galagidae**

*Galago senegalensis –* ZMB 64278, ZMB 3812, ZMB 64218

*Otolemur garnettii –* ZMB 64242, ZMB 64282

*Euoticus elegantulus –* ZMB 75692, ZMB 63728

**Lorisidae**

*Loris tardigradus –* ZMB 20822, ZMB 91344

*Nycticebus coucang –* ZMB 34262, ZMB 105769, ZMB 809

*Perodicticus potto –* ZMB 86040, ZMB 23441

*Arctocebus calabarensis –* ZMB 12081, ZMB 84319

**Tarsioidea**

*Tarsius spectrum –* ZMB 91447, ZMB 5017

*Tarsius syrichta –* ZMB 5306

**Callitrichinae**

*Cebuella pygmaea –* ZMB 5655

*Callithrix jacchus –* ZMB 36453, ZMB 36449, ZMB 36436

*Leontopithecus rosalia –* ZMB 300, ZMB 301

*Leontopithecus chrysopygus –* ZMB 304, ZMB 305

*Saguinus mystax –* ZMB 45036, ZMB 35796

*Saguinus oedipus* – ZMB 296, ZMB 297

***Aotinae***

*Aotus sp. –* ZMB 3188, ZMB 13154, ZMB 13159, ZMB 35783, ZMB 35784, ZMB 35791, ZMB 35792, ZMB 35793, ZMB 38430, ZMB 91372, ZMB 91373

**Cebinae**

*Saimiri sp.–* ZMB 35779, ZMB A4168

*Cebus olivaceus –* ZMB 225

*Cebus albifrons –* ZMB 228, ZMB 229, ZMB 15586, ZMB 16647

*Cebus capucinus –* ZMB 233

*Sapajus apella* – ZMB 7888, ZMB 37438, ZMB 40114, ZMB 49398, ZMB 90688, ZMB 90692

**Pitheciinae**

*Cacajao calvus –* ZMB 18480, ZMB 43637, ZMB 45251

*Cacajao melanocephalus –* ZMB 35311, ZMB 43635, ZMB 90744, ZMB 35311, ZMB 43635

*Chiropotes satanas –* ZMB 85177, ZMB 16485, ZMB 10325, ZMB 252, ZMB 251, ZMB A2951, ZMB 10325, ZMB 252, ZMB A2951

*Pithecia pithecia –* ZMB 246, ZMB 247, ZMB 38456, ZMB 33837, ZMB 33937, ZMB 35770

**Callicebinae**

*Callicebus moloch* – ZMB 258, ZMB 261, ZMB 85180, ZMB 85181, ZMB 85182

**Atelinae**

*Ateles sp*. *–* ZMB 7754, ZMB 375, ZMB 640, ZMB 832, ZMB 7754, ZMB 7947, AMNH 11074, AMNH 17220, AMNH 17222, ANMH 29844

*Alouatta sp.*– ZMB 29420, ZMB 34287, ANMH 23347, ANMH 32145, ANMH 33063, ANMH 33074, ANMH 73548, ANMH 73553

**Cercopithecini**

*Erythrocebus patas* – ZMB 11864, ZMB 87490

*Chlorocebus aethiops* – ZMB 87509, ZMB 87476, ZMB 6463

*Miopithecus talapoin* – ZMB 4943, ZMB 19083

*Cercopithecus mitis* – ZMB 87536, ZMB 87895, ZMB 87876, ZMB 87850, ZMB 87754, ZMB 4102

**Papionini**

*Macaca fascicularis* – ZMB 12557, ZMB 108, ZMB 110, ZMB 113, ZMB 111, ZMB 118, ZMB 1255, ZMB 7764, ZMB 5443, ZMB 7702, ZMB 49096, ZMB 43867, ZMB 49091, ZMB 48463, ZMB 48469, ZMB 48499, ZMB 48500, ZMB 49073, ZMB 49086, ZMB 49093, ZMB 49094, WFU 1763, WFU 1278, ZMB 7070,

*Macaca fuscata* – PRI 2868, PRI 3337, PRI 4373, PRI 5743, PRI 3280, PRI 3281, PRI 4325, PRI 5160, PRI 5288, PRI 5189, PRI 5190

*Macaca arctoides* – ZMB 12317, ZMB 12404

*Macaca sylvanus* – ZMB 566, ZMB 14445

*Lophocebus albigena* – ZMB 18528, ZMB 23243, ZMB 72145, ZMB 72155, ZMB A60.06

*Papio anubis* – ZMB 75019, ZMB 7589, ZMB 11175, ZMB 11199, ZMB 11555, ZMB 12386, ZMB 12832, ZMB 18797, ZMB 20852, ZMB 74616, ZMB 74762, ZMB 74918, ZMB 74955, ZMB 74990, ZMB 75013

*Theropithecus gelada* – ZMB 72126, ZMB 85753

*Mandrillus sphinx* – ZMB, 7959, ZMB 11183, ZMB 7696

*Mandrillus leucophaeus* – ZMB 2.1.94

*Cercocebus torquatus* – ZMB 15776, ZMB 16488

**Colobinae**

*Nasalis larvatus* – ZMB 72215, ZMB 72980, ZMB 73073

*Semnopithecus entellus* – ZMB 13701, ZMB 16107

*Trachypithecus cristatus –* ZMB 43652, ZMB 42747, ZMB 42606, ZMB 43652, ZMB 72975

*Trachypithecus vetulus –* ZMB 28, ZMB 73059

*Presbytis comata –* ZMB 48421

*Presbytis melalophos –* ZMB 73048, ZMB 73024, ZMB 73017, ZMB 73025,

*Piliocolobus pennantii –* ZMB 5873, ZMB 4191

*Colobus guereza –* ZMB 45404, ZMB 30112, ZMB 86811, ZMB 86858, ZMB 86915, ZMB 86949, ZMB 87060, ZMB 87191

**Hominidae**

*Pan troglodytes –* MRAC 178, MRAC 929, MRAC 10447, MRAC 10733, MRAC 10800, MPI 11800, MPI 15008, MPI 11776, MPI 11778, MPI 11779, MPI 11784, MPI 11789, MPI 11790, MPI 11791, MPI 11792, MPI 11796, MPI 11798, MPI 11800, MPI 11903, MPI 12176, MPI 13433, MPI 13437, MPI 13438, MPI 13439, MPI 14992, MPI 14994, MPI 15008, MPI 15013, ZMB 83641, ZMB 15849, ZMB 16968, ZMB 17011, ZMB 24838, ZMB 27054, ZMB 30846, ZMB 30847, ZMB 32356, ZMB 35526, ZMB 46095, ZMB 47506, ZMB 6983, ZMB 72844, ZMB 7537, ZMB 83597, ZMB 83604, ZMB 83610, ZMB 83619, ZMB 83623, ZMB 83635, ZMB 83639, ZMB 83661, ZMB 83673, ZMB 83685, ZMB 83830, ZMB A162.07

*Pan paniscus –* MRAC 9369, MRAC 22908, MRAC 27009, MRAC 29010, MRAC 29016, MRAC 29024, MRAC 29026, MRAC 29029, MRAC 29030, MRAC 29033, MRAC 29041, MRAC 29048, MRAC 29055, MRAC 29056, MRAC 29066, MRAC 84036M02, MRAC 84036M03, MRAC 84036M02, MRAC 84036M04, MRAC 84036M09, MRAC 84036M10, MRAC 84036M11

*Homo sapiens* – RIA 433, ULAC 1, ULAC 1002, ULAC 101, ULAC 1079, ULAC 119, ULAC 13, ULAC 140, ULAC 151, ULAC 171, ULAC 179, ULAC 259, ULAC 264, ULAC 477, ULAC 522, ULAC 536, ULAC 566, ULAC 58, ULAC 607, ULAC 659, ULAC 66, ULAC 738, ULAC 74, ULAC 742, ULAC 752, ULAC 790, ULAC 797, ULAC 799_27, ULAC 799_28, ULAC 801, ULAC 806, ULAC 81, ULAC 83, ULAC 864, ULAC 921, ULAC 966, ULAC 982, ULAC 997, NMNH SI12, NMNH SI13, NMNH SI17, NMNH SI18, NMNH SI3, NMNH SI34, NMNH SI35, NMNH SI36, NMNH SI37, NMNH SI38, NMNH SI4, NMNH SI40, NMNH SI42, NMNH SI44, NMNH SI45, NMNH SI46, NMNH SI47, NMNH SI48,

*Gorilla gorilla –* ZMB 17963, ZMB 30940, ZMB 30941, ZMB 31277, ZMB 31435, ZMB 31426, ZMB 83545, ZMB 83546, ZMB 83551, ZMB 83561, ZMB 83581, NMNH 545037,

*Pongo pygmaeus –* UGAZ 14.5.10, UGAZ 14.5.8, SMF/CA243, SMF/CA260, SMF/CA378, SMF/CA539, SMF 541, SMF 602, SMF 621, SMF 679, SMF 687, SMF 795, SMF 827, SMF 83, SMF 87, SMF 1113, SMF 1577, SMF 15837, SMF 2639, SMF 2654, SMF 38296, SMF 59140, SMF 59141, SMF 59142, SMF 59153, SMF 59157, SMF 74303, ZMB 12209, ZMB 30944, ZMB 30946, ZMB 38607, ZMB 67173, ZMB 6948, ZMB 6954, ZMB 6957, ZMB 6987, ZMB 7875, ZMB 83509, ZMB 83511, ZMB 83515

**Hylobatidae**

*Hylobates muelleri –* AMNH 103725, AMNH 103726, AMNH 106781, ZMB 7814

*Hylobates agilis –* ZMB 38562, ZMB 35368

*Symphalangus syndactylus –* AMNH 100048, AMNH 102193

*Hoolock sp. –* ZMB 7837

**Institutes**

**ZMB** – Museum für Naturkunde, Leibniz-Institut für Evolutions- und Biodiversitätsforschung an der Humboldt-Universität zu Berlin, Invalidenstraße 43, 10115 Berlin, Germany

**WFU –** Wake Forest University Primate Centre, Winston-Salem, NC, United States

**PRI –** Kyoto University Primate Research Institute, Inuyama, Japan

**AMNH –** American Museum of Natural History, 79 Street and Central Park West, New York, NY 10024, United States

**MRAC –** Musee Royal de l'Afrique Centrale (MRAC), Leuvensesteenweg 13, 3080 Tervuren, Belgium

**MPI –** Max Planck Institute for Evolutionary Anthropology, Department of Primatology, Deutscher Platz 6, 04103 Leipzig, Germany

**RIA –** Institutul de Antropologie “Francisc J. Rainer” (FJR), Bd. Eroii Sanitari nr. 8, C. P. 35-13, Bucureşti, cod 050474, România

**ULAC –** Universität Leipzig, Institut für Anatomie, Lehrsammlung Anatomie, Liebigstraße 13, 04103 Leipzig

**NMNH –** National Museum of Natural History (NMNH), P.O. Box 37012 Smithsonian Inst., Washington D.C., 20013-7012

**UGAZ –** Institut für Anatomie und Zellbiologie (Ernst-Moritz-Arndt-Universität Greifswald), Friedrich-Loeffler-Str. 23c, 17489 Greifswald, Germany

**SMF -** Forschungsinstitut Senckenberg (SMF), Research Institute and Natural History Museum, Senckenberganlage 25, 60325 Frankfurt am Main, Germany
