# Supplementary material for: Primate tooth crown nomenclature revisited
Source: PeerJ. 2023 Jan 12;11:e14523. doi: 10.7717/peerj.14523 (PMC9840859; doi:10.7717/peerj.14523)
Supplement: File S2 [file peerj-11-14523-s002.pdf]

C)

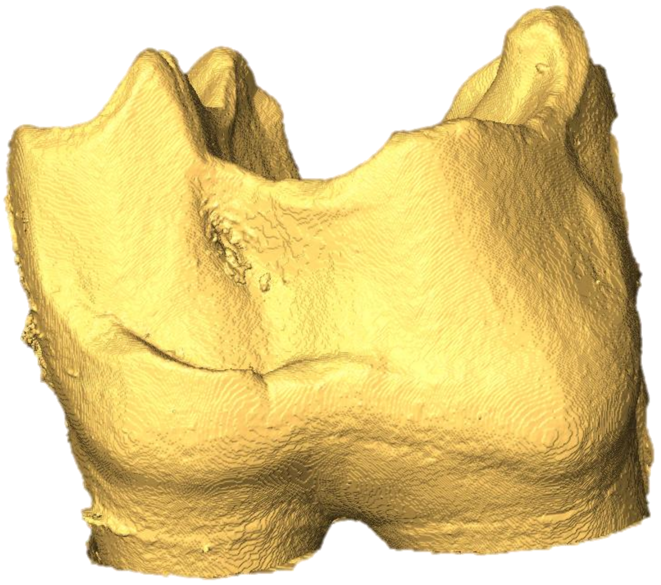

Hapelemur griseus – ZMB 43240 LLM2

D)

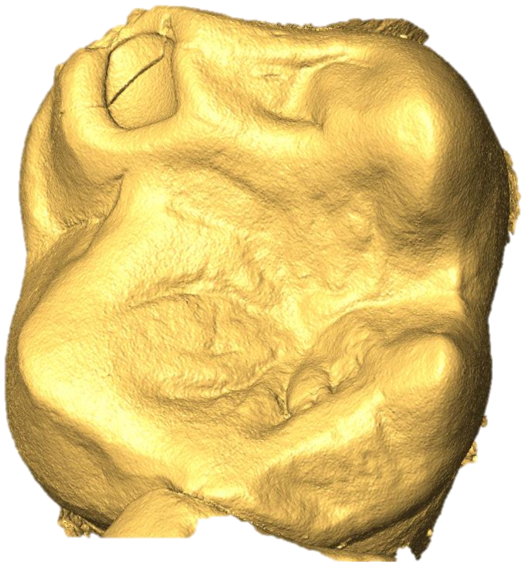

Prolemur simus – ZMB 5511 LLM2

E)

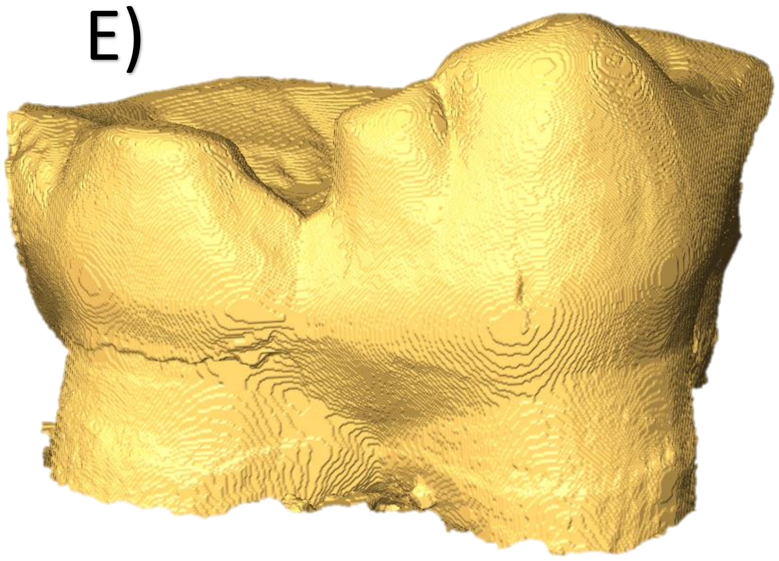

Prolemur simus – ZMB 5511 LLM1

F)

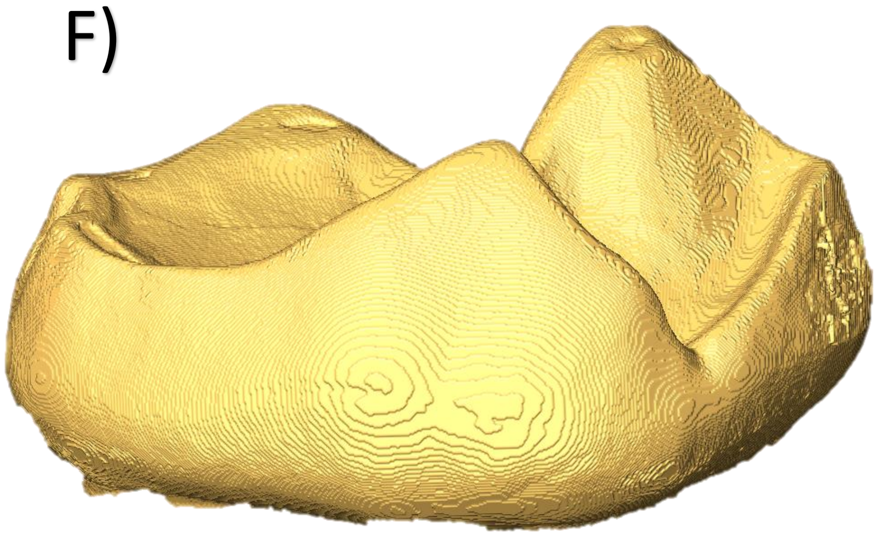

Eulemur fulvus – ZMB 44476 LLM2

G)

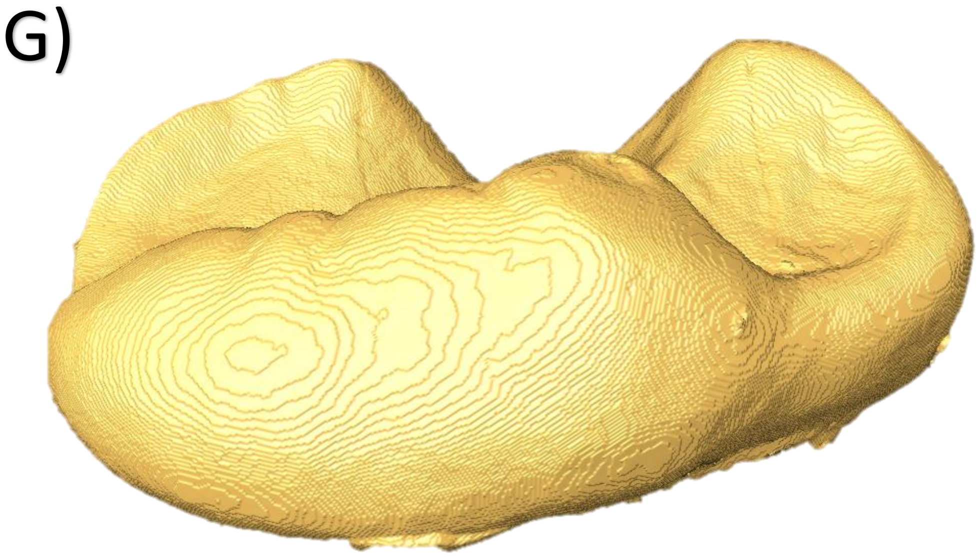

Varecia variegate – ZMB MAM_A LLM2

C)

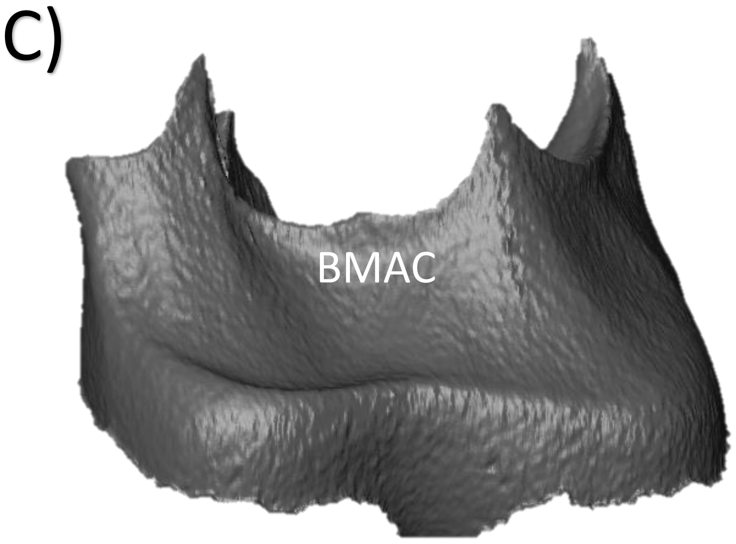

Hapelemur griseus – ZMB 43240 LLM2

D)

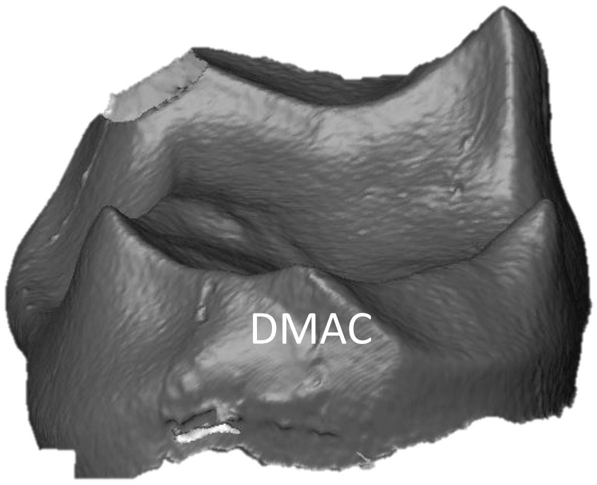

Prolemur simus – ZMB 5511 LLM2

E)

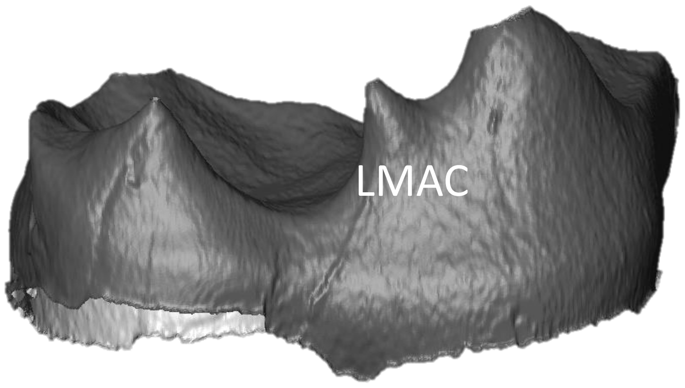

Prolemur simus – ZMB 5511 LLM1

F)

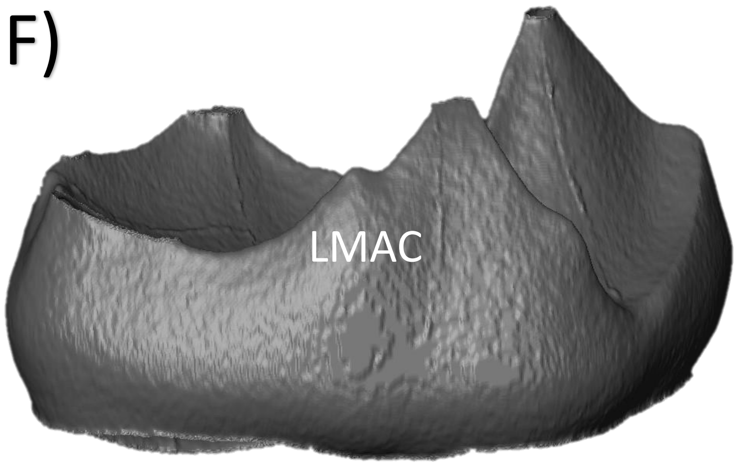

Eulemur fulvus – ZMB 44476 LLM2

G)

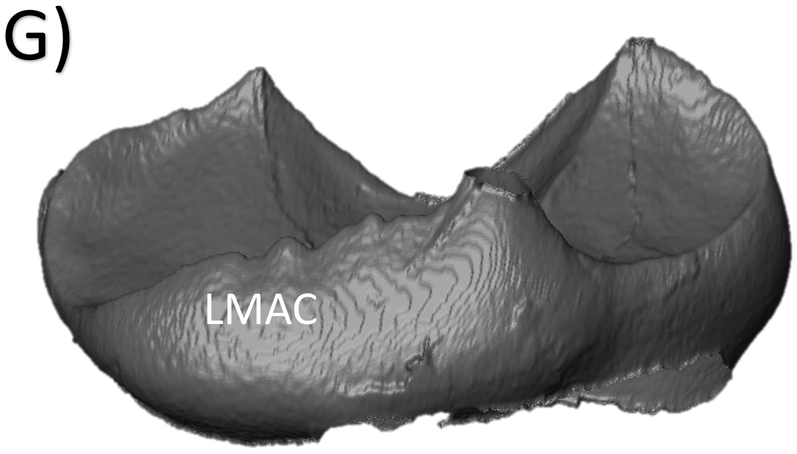

Varecia variegate – ZMB MAM_A LLM2

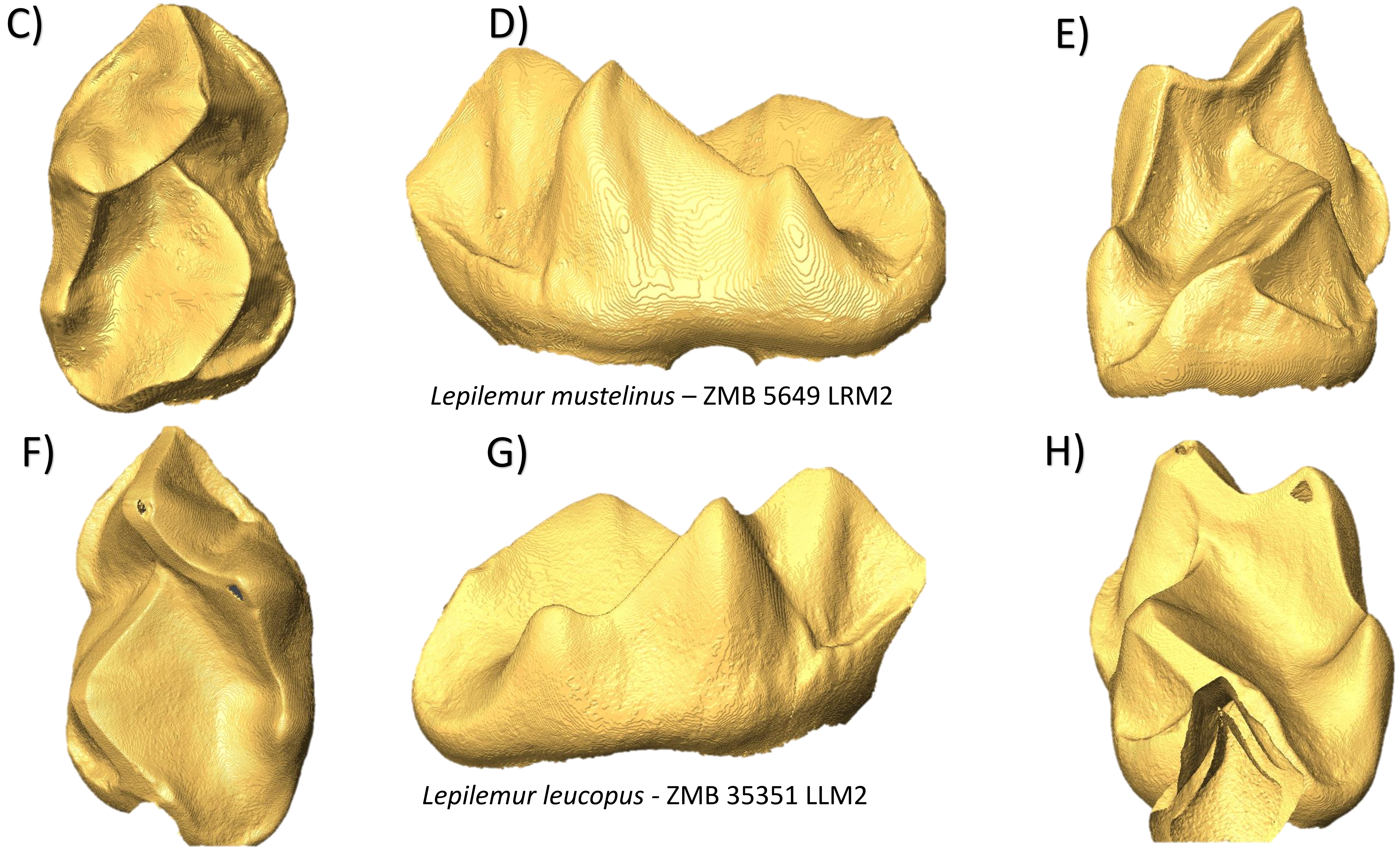

C)
D)
E)
Lepilemur mustelinus – ZMB 5649 LRM2
F)
G)
H)
Lepilemur leucopus - ZMB 35351 LLM2

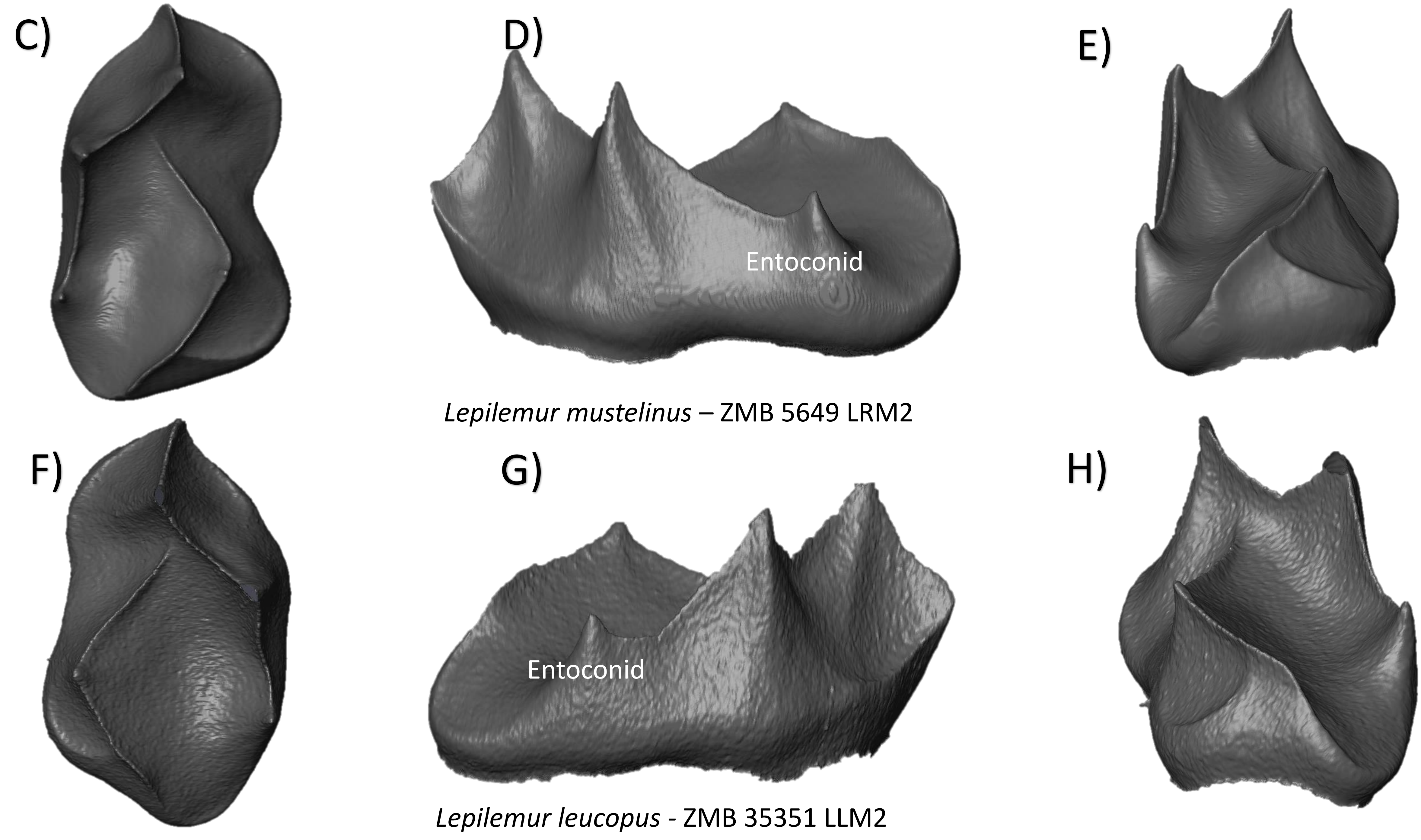

C)
D)
E)
Entoconid
Lepilemur mustelinus – ZMB 5649 LRM2
F)
G)
H)
Entoconid
Lepilemur leucopus - ZMB 35351 LLM2

C)

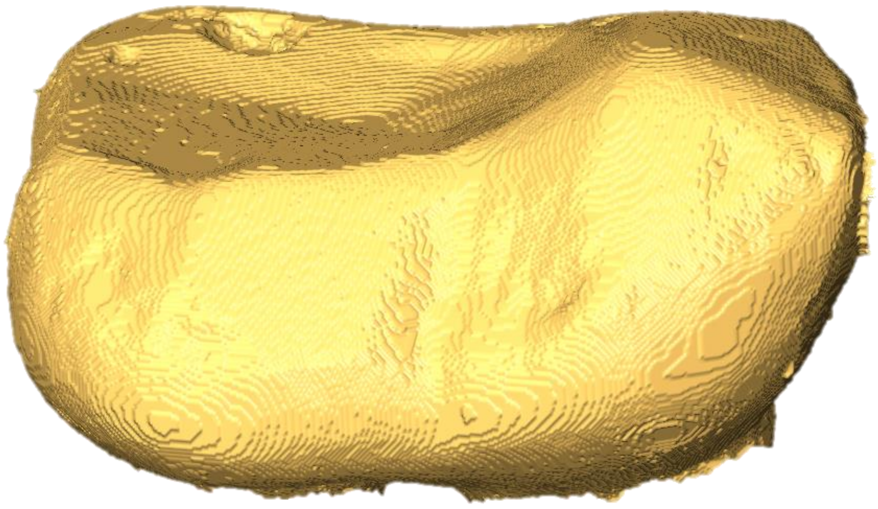

Cheirogaleus sp. – ZMB 4823 LRM2

D)

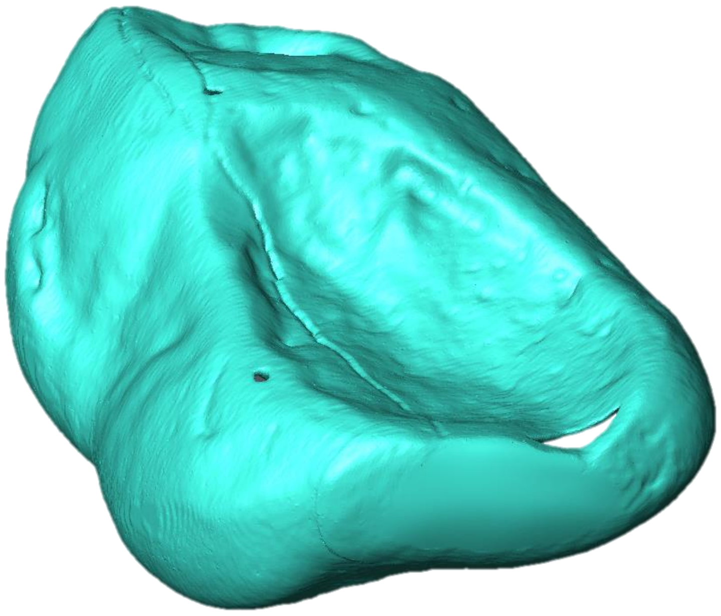

Cheirogaleus sp. – ZMB 35352 LLM1

E)

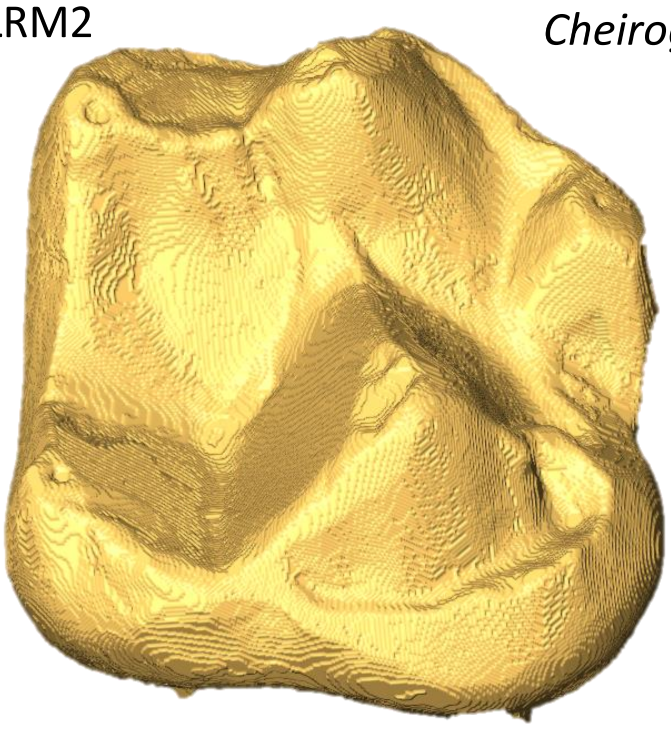

Phaner furcifer – ZMB 3838 LRM2

C)

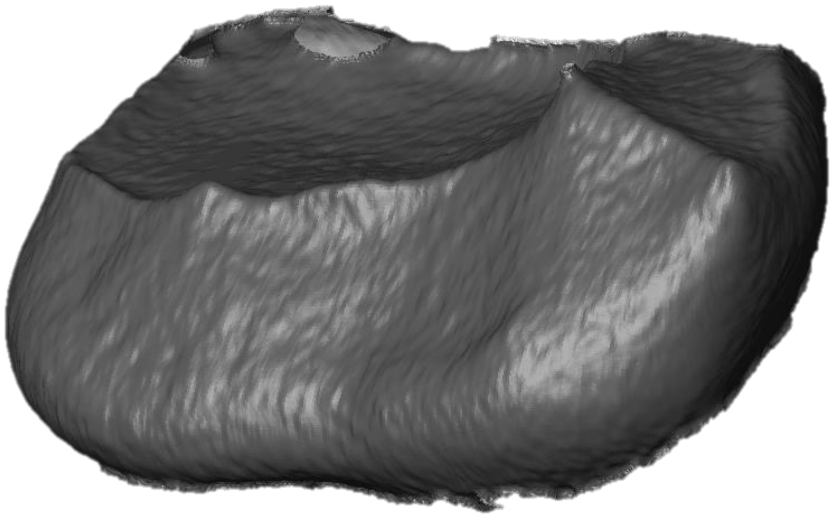

Cheirogaleus sp. – ZMB 4823 LRM2

D)

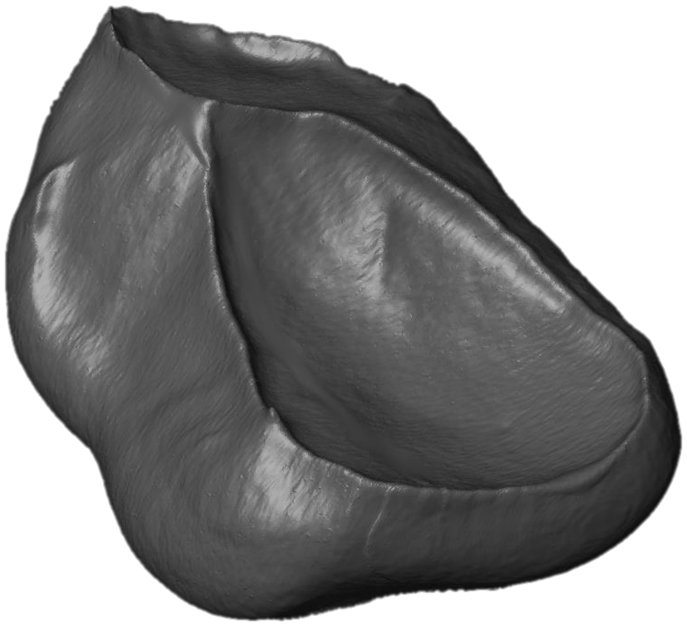

Cheirogaleus sp. – ZMB 35352 LLM1

E)

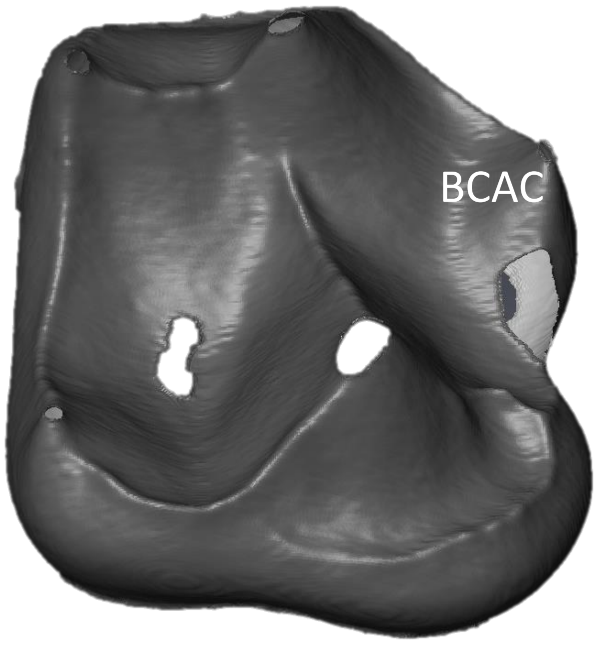

Phaner furcifer – ZMB 3838 LRM2

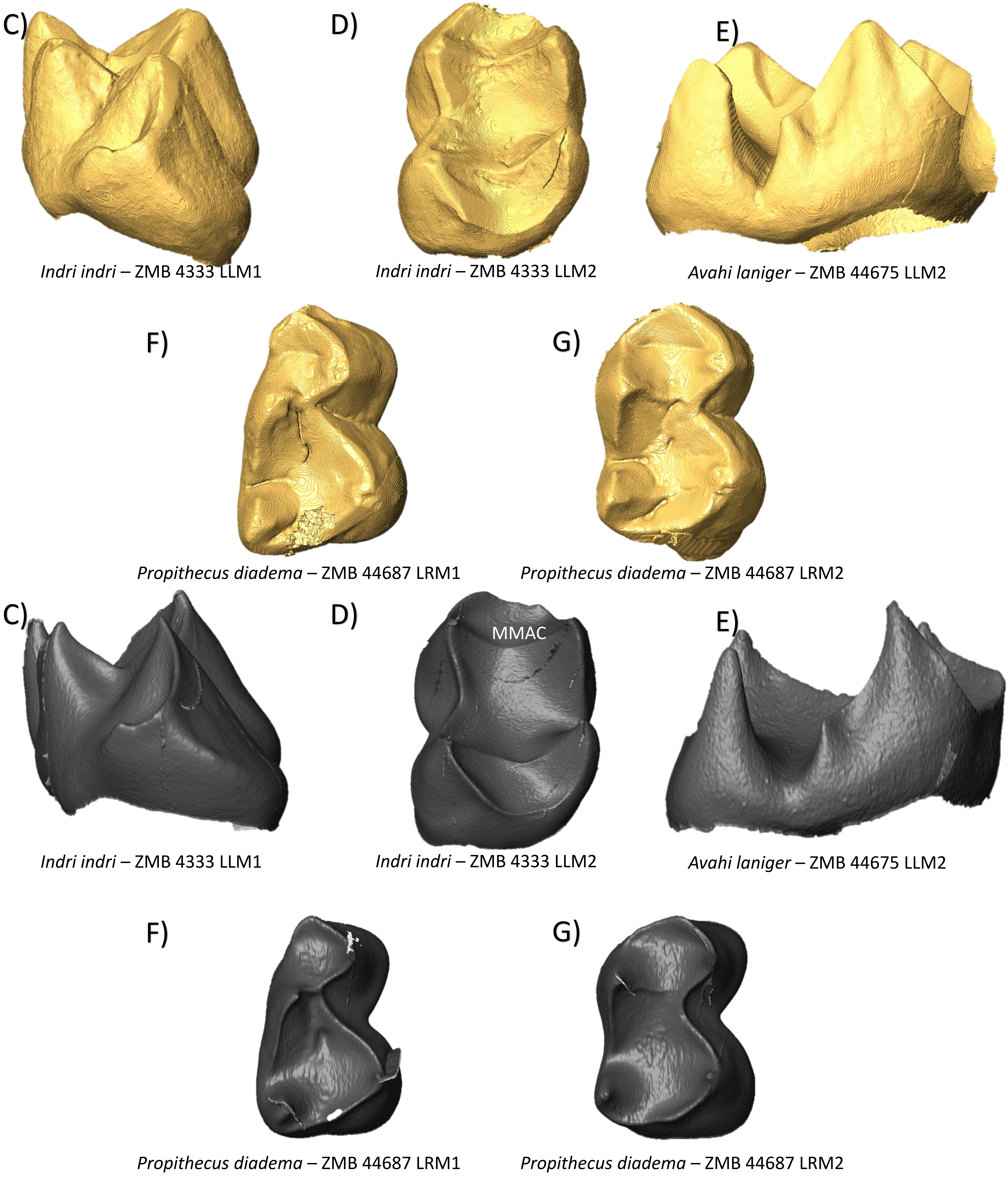
C)
D)
E)
Indri indri – ZMB 4333 LLM1
Indri indri – ZMB 4333 LLM2
Avahi laniger – ZMB 44675 LLM2
F)
G)
Propithecus diadema – ZMB 44687 LRM1
Propithecus diadema – ZMB 44687 LRM2
C)
D)
MMAC
E)
Indri indri – ZMB 4333 LLM1
Indri indri – ZMB 4333 LLM2
Avahi laniger – ZMB 44675 LLM2
F)
G)
Propithecus diadema – ZMB 44687 LRM1
Propithecus diadema – ZMB 44687 LRM2

C)

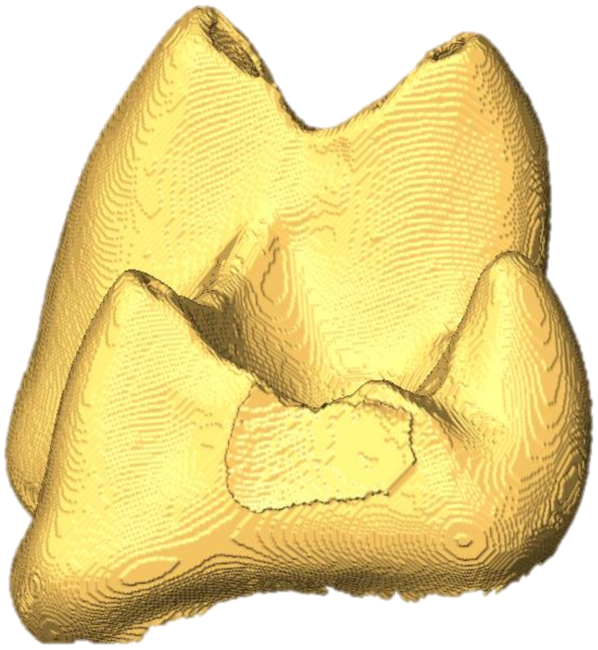

Galago senegalensis – ZMB 64278 LLM1

D)

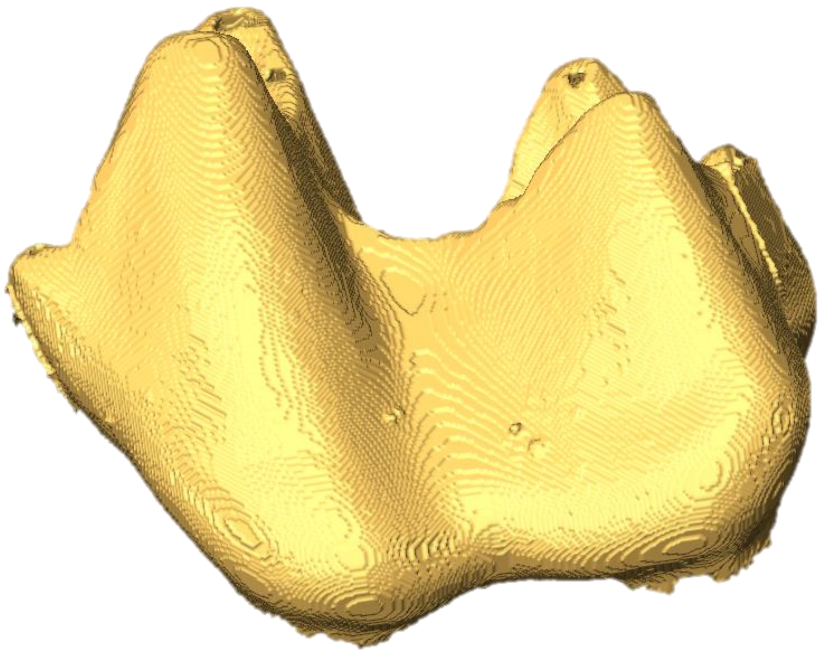

Galago senegalensis – ZMB 64278 LLM1

E)

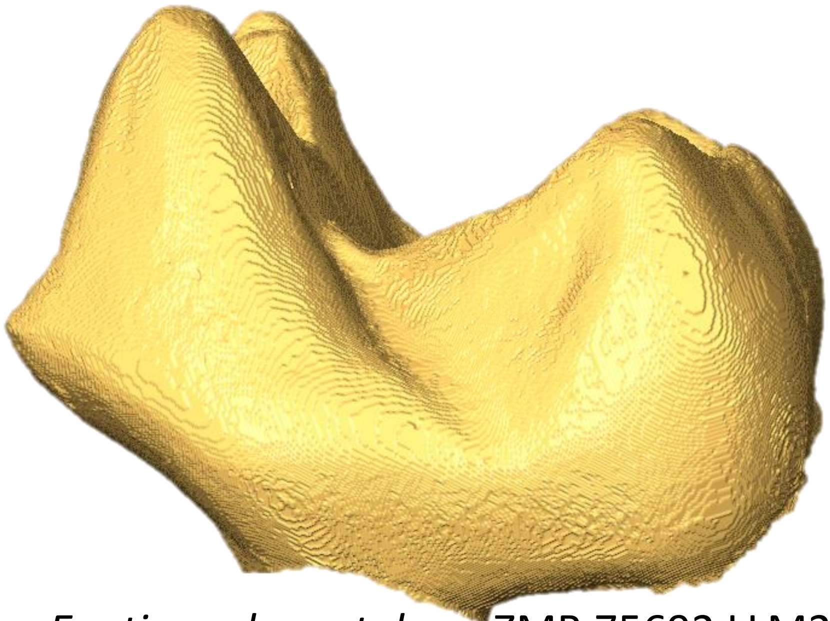

Euoticus elegantulus – ZMB 75692 LLM2

F)

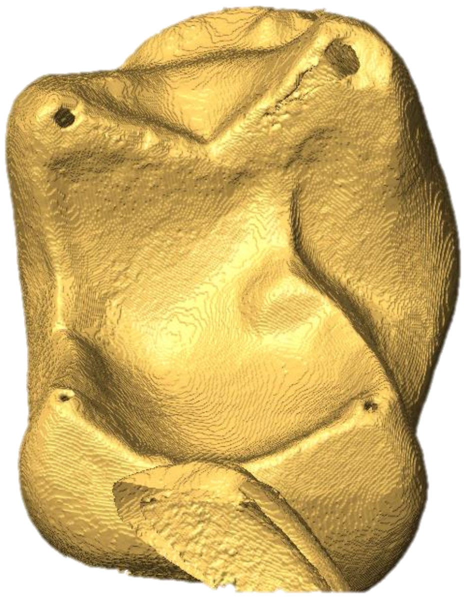

Euoticus elegantulus – ZMB 63728 LRM2

C)

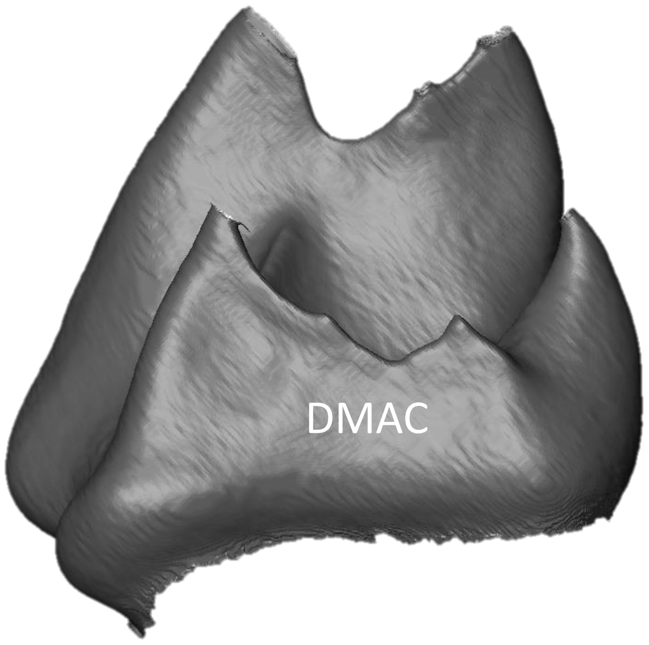

Galago senegalensis – ZMB 64278 LLM1

D)

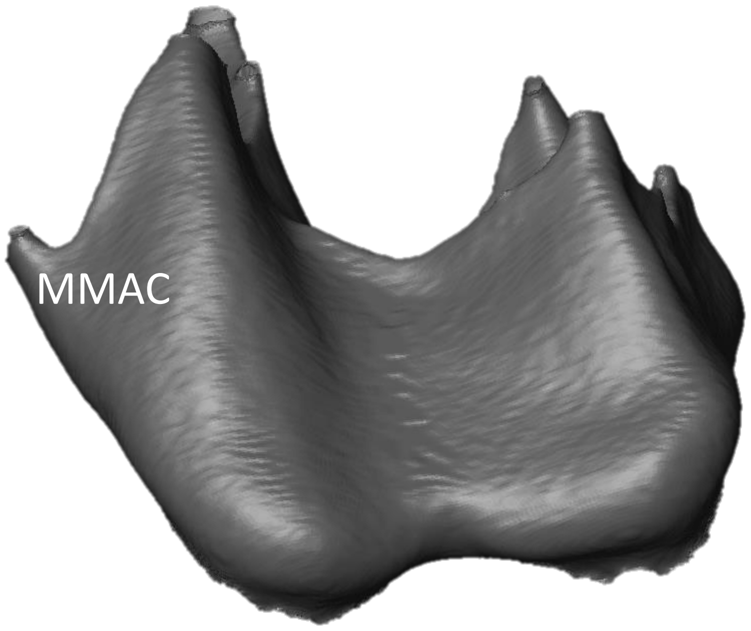

Galago senegalensis – ZMB 64278 LLM1

E)

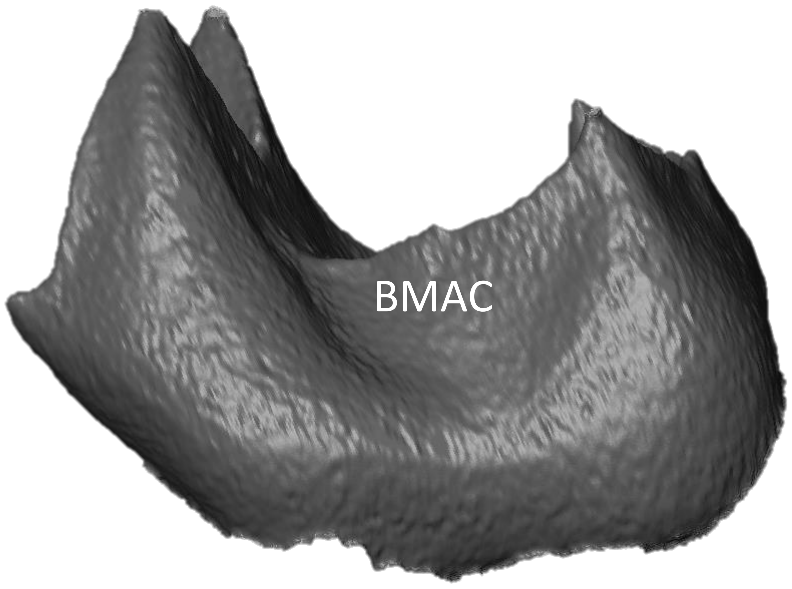

Euoticus elegantulus – ZMB 75692 LLM2

F)

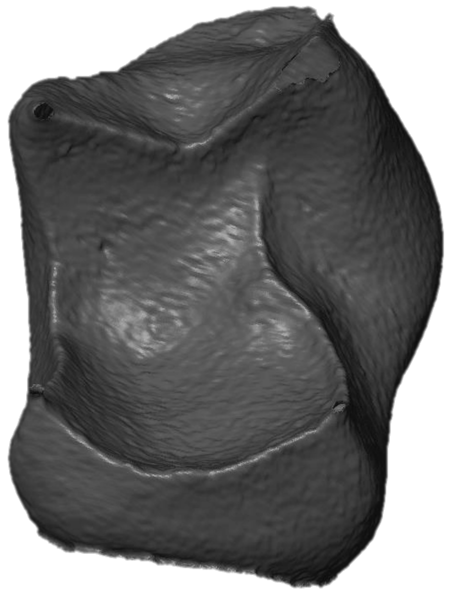

Euoticus elegantulus – ZMB 63728 LRM2

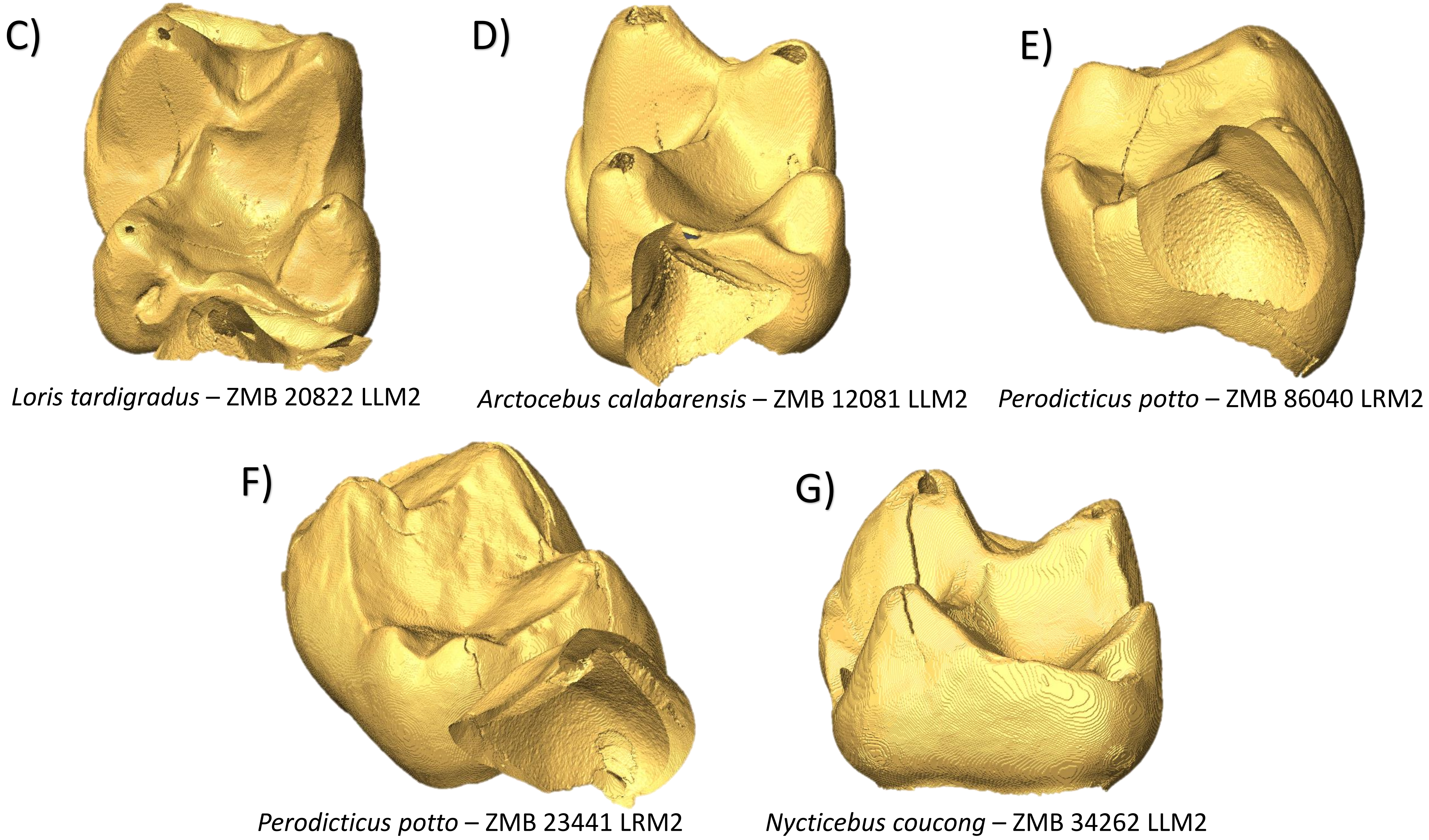
C)
D)
E)
Loris tardigradus – ZMB 20822 LLM2
Arctocebus calabarensis – ZMB 12081 LLM2
Perodicticus potto – ZMB 86040 LRM2
F)
G)
Perodicticus potto – ZMB 23441 LRM2
Nycticebus coucong – ZMB 34262 LLM2

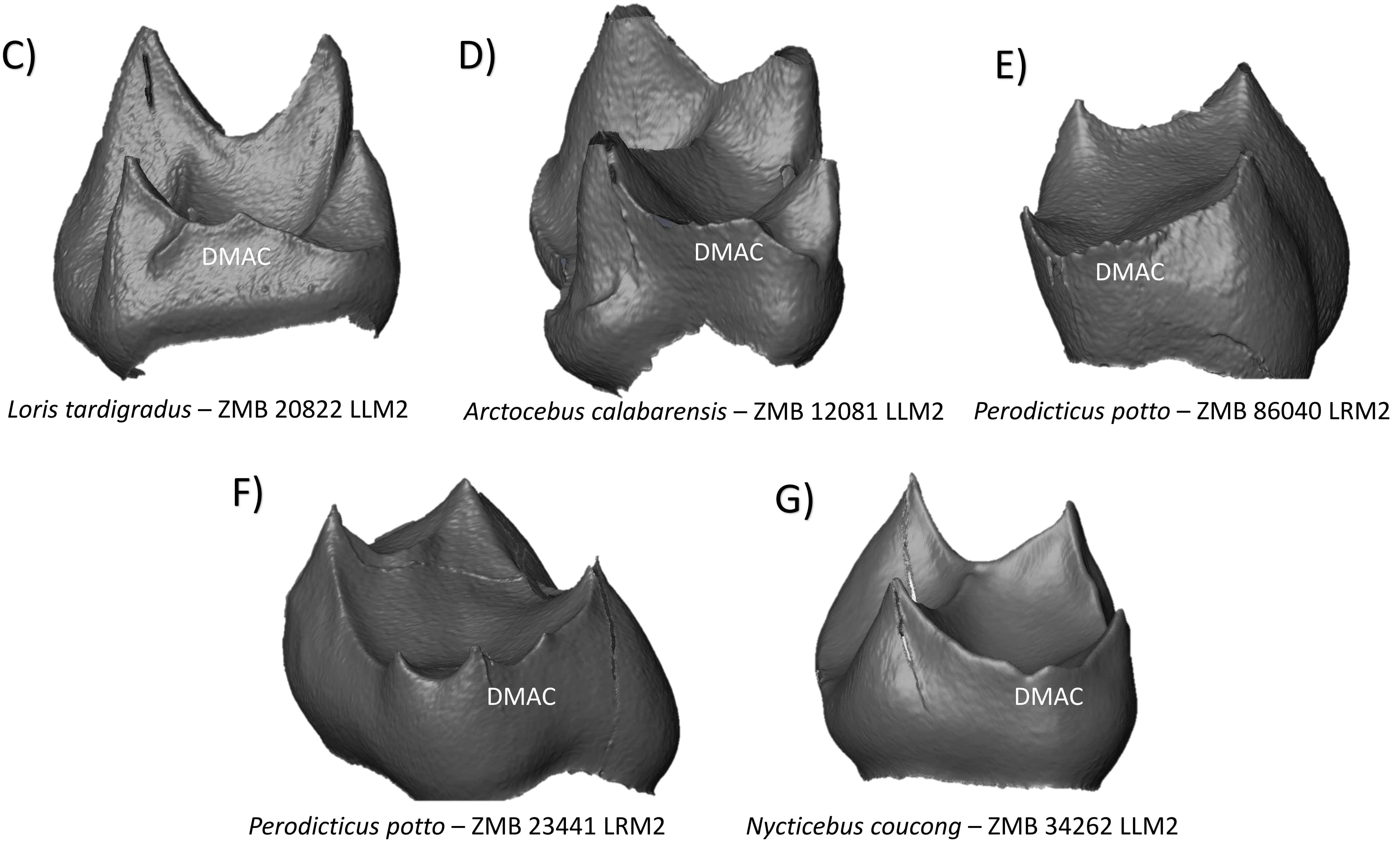
C)
D)
E)
DMAC
DMAC
DMAC
Loris tardigradus – ZMB 20822 LLM2
Arctocebus calabarensis – ZMB 12081 LLM2
Perodicticus potto – ZMB 86040 LRM2
F)
G)
DMAC
DMAC
Perodicticus potto – ZMB 23441 LRM2
Nycticebus coucong – ZMB 34262 LLM2

C)

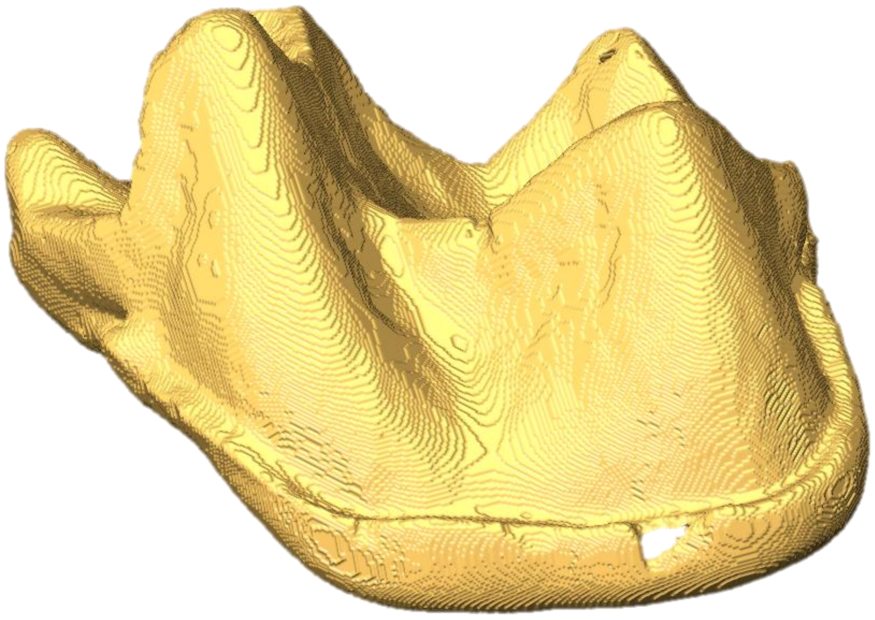

Tarsius sprectrum – ZMB 91447 LLM1

D)

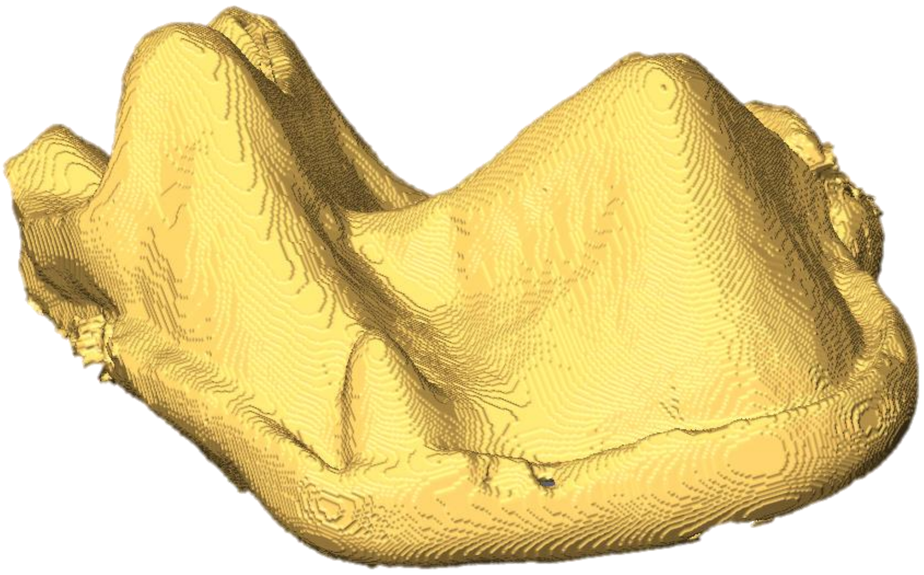

Tarsius sprectrum – ZMB 91447 LLM2

E)

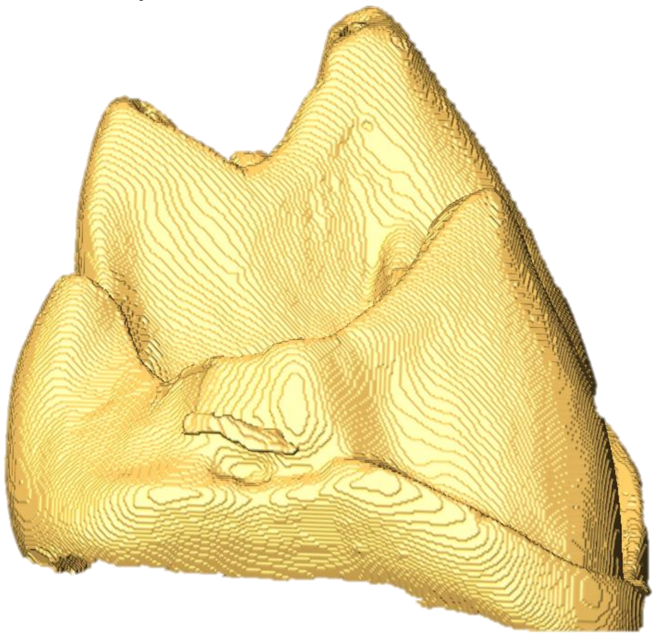

Tarsius sprectrum – ZMB 5017 LRM1

F)

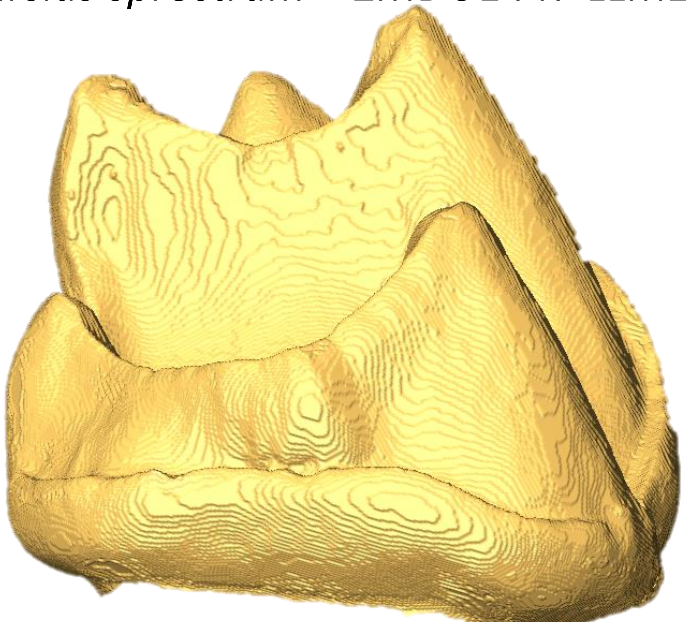

Tarsius syrichta – ZMB 5306 LRM2

C)

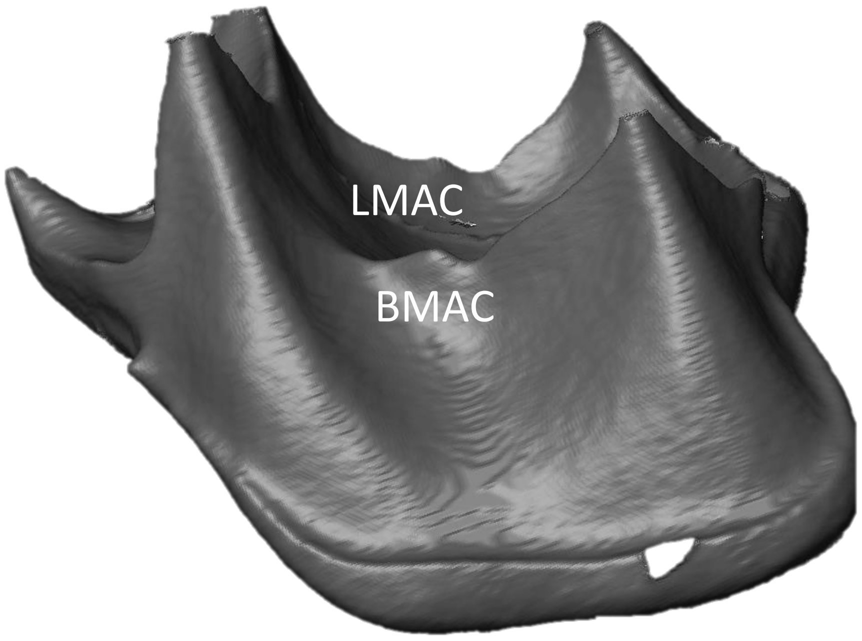

Tarsius sprectrum – ZMB 91447 LLM1

D)

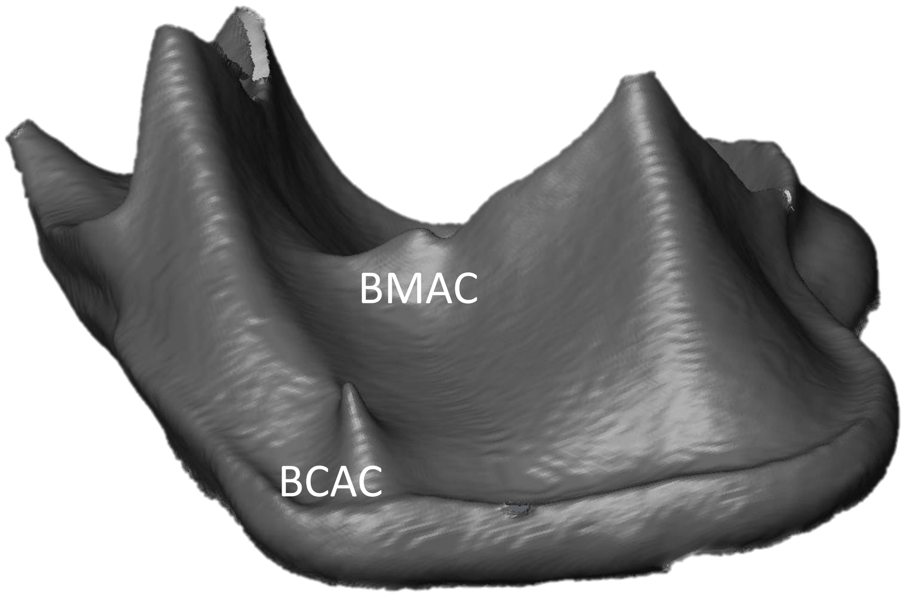

Tarsius sprectrum – ZMB 91447 LLM2

E)

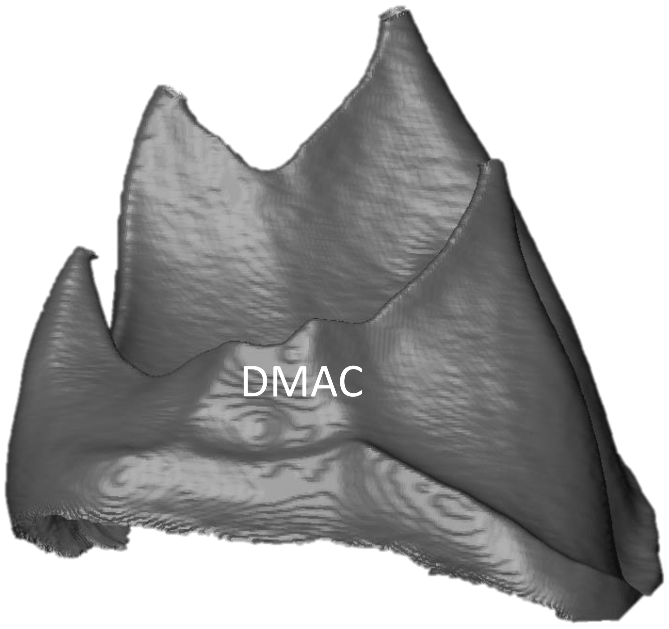

Tarsius sprectrum – ZMB 5017 LRM1

F)

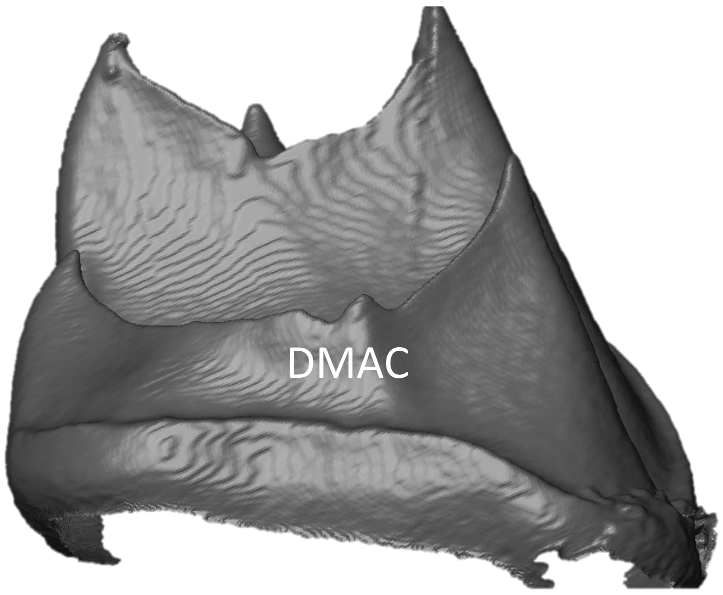

Tarsius syrichta – ZMB 5306 LRM2

C)

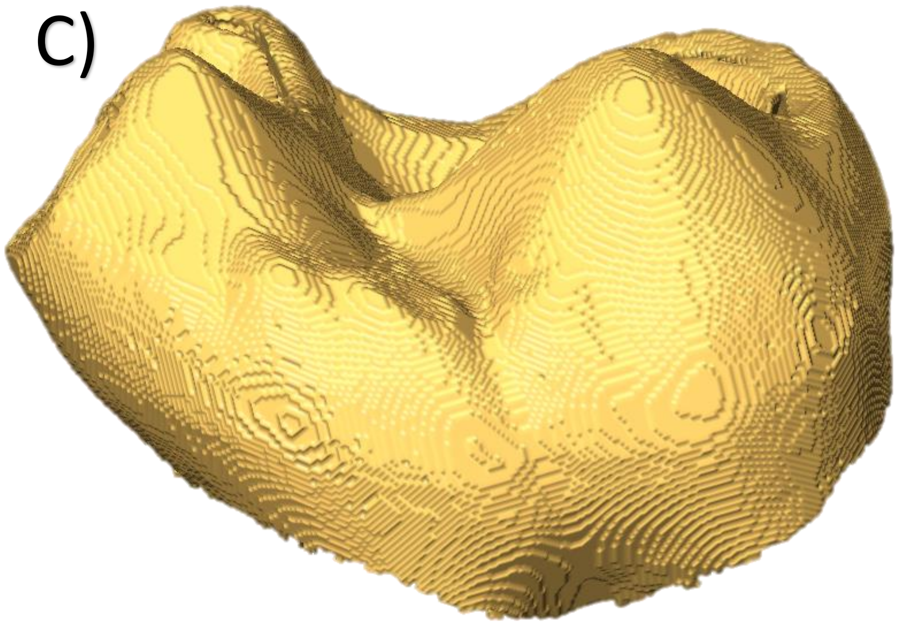

Cebulla pygmaea – ZMB 5655 LLM1

D)

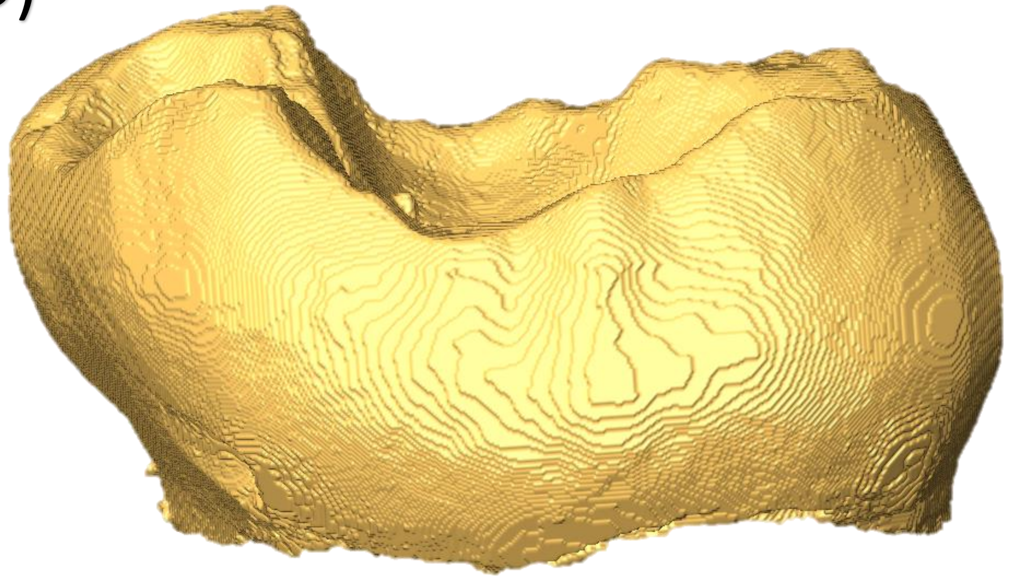

Leontopithecus rosalia – ZMB 300 LRM2

E)

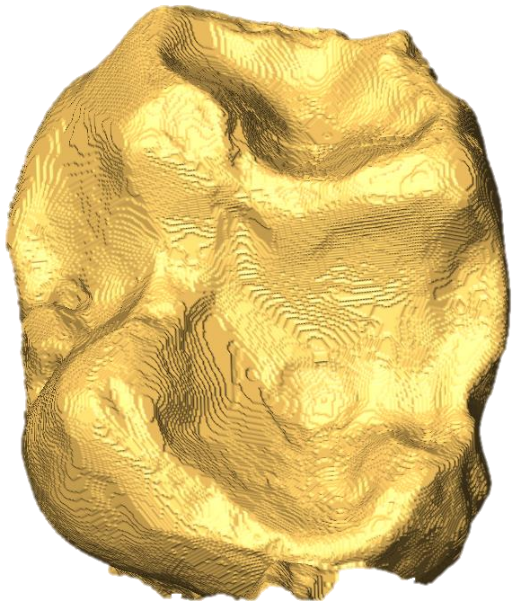

Saguinas oedipus – ZMB 297 LLM1

F)

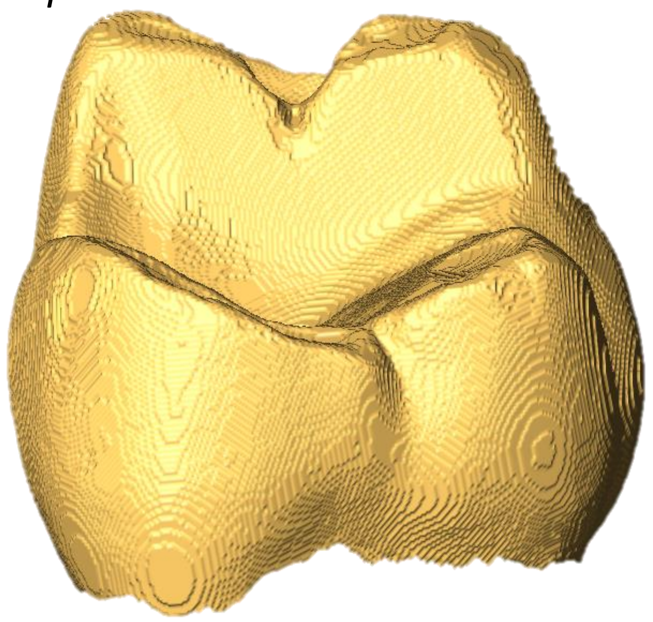

Callithrix jacchus – ZMB 36453 LRM2

C)

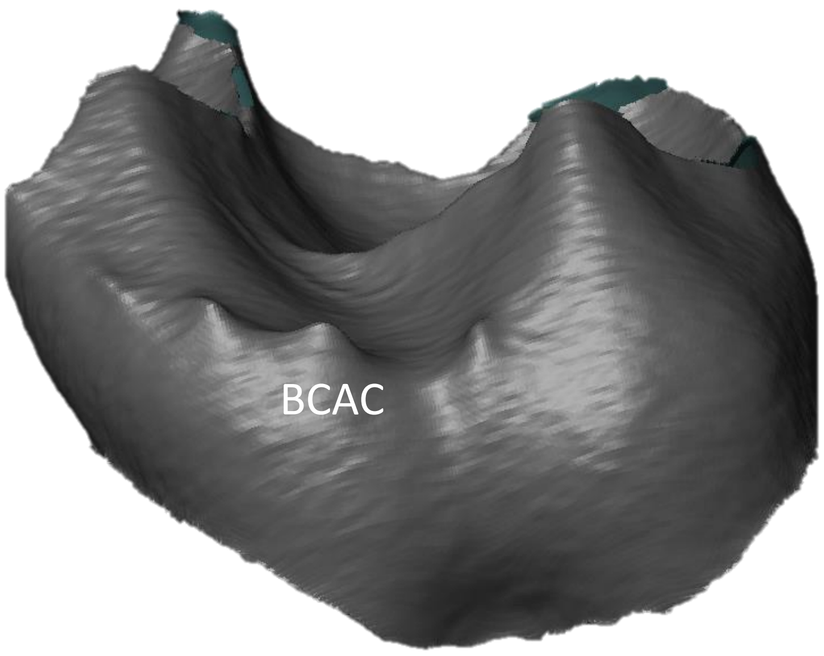

Cebulla pygmaea – ZMB 5655 LLM1

D)

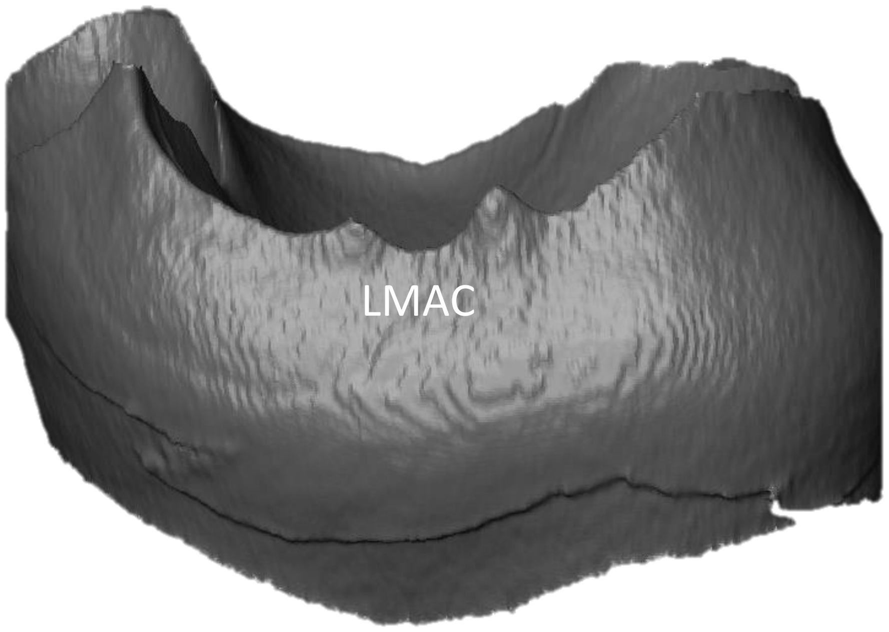

Leontopithecus rosalia – ZMB 300 LRM2

E)

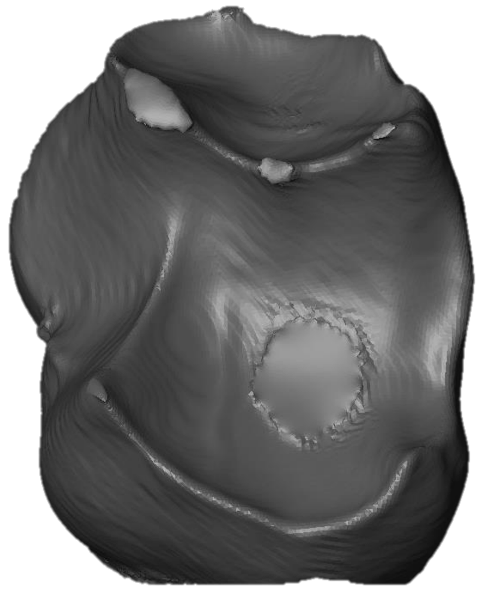

Saguinas oedipus – ZMB 297 LLM1

F)

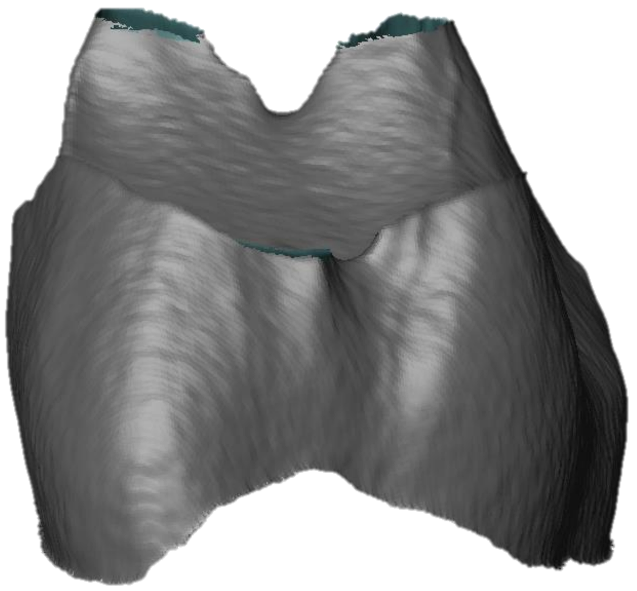

Callithrix jacchus – ZMB 36453 LRM2

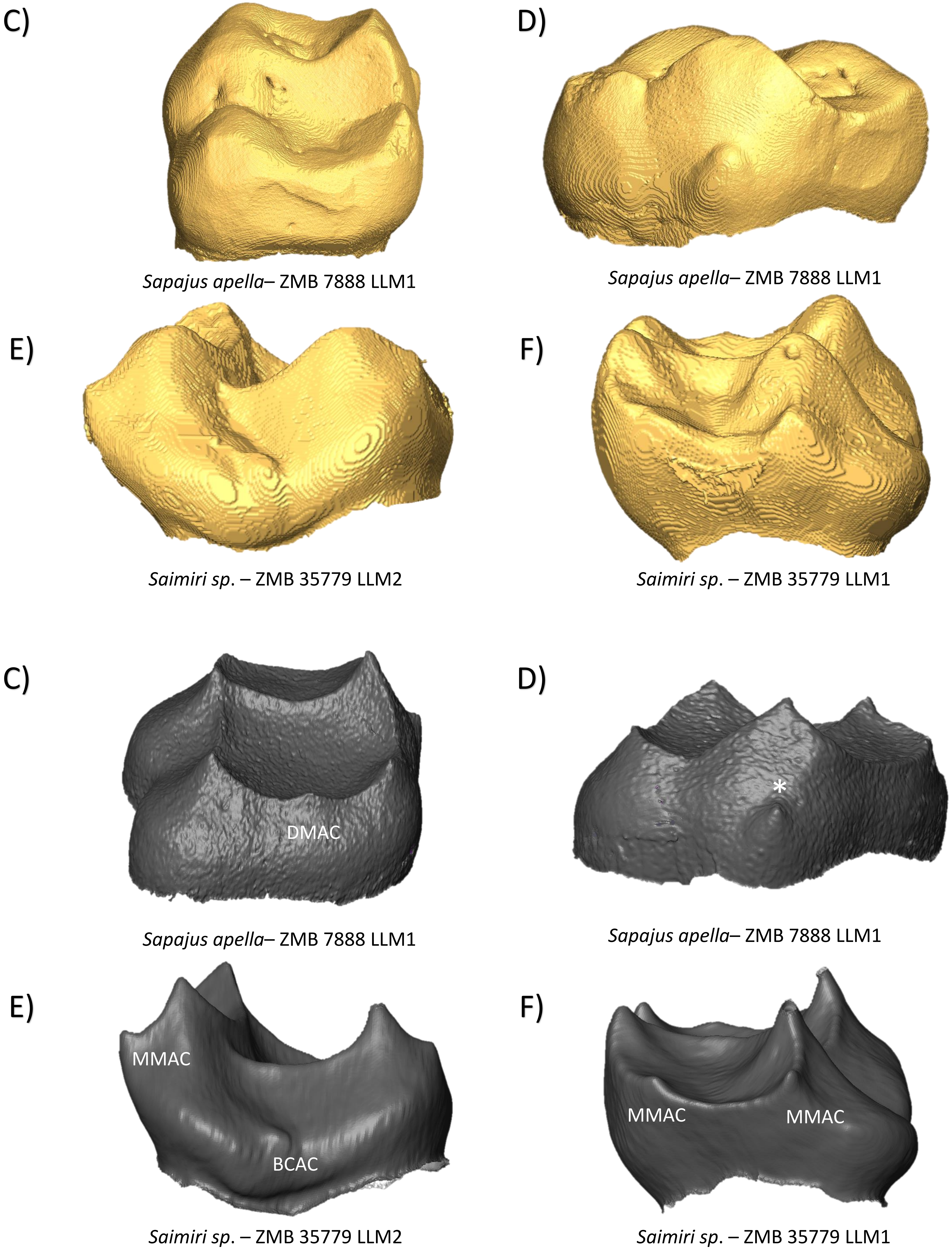
C)
Sapajus apella– ZMB 7888 LLM1
D)
Sapajus apella– ZMB 7888 LLM1
E)
Saimiri sp. – ZMB 35779 LLM2
F)
Saimiri sp. – ZMB 35779 LLM1
C)
DMAC
Sapajus apella– ZMB 7888 LLM1
D)
*
Sapajus apella– ZMB 7888 LLM1
E)
MMAC
BCAC
Saimiri sp. – ZMB 35779 LLM2
F)
MMAC
MMAC
Saimiri sp. – ZMB 35779 LLM1

C)

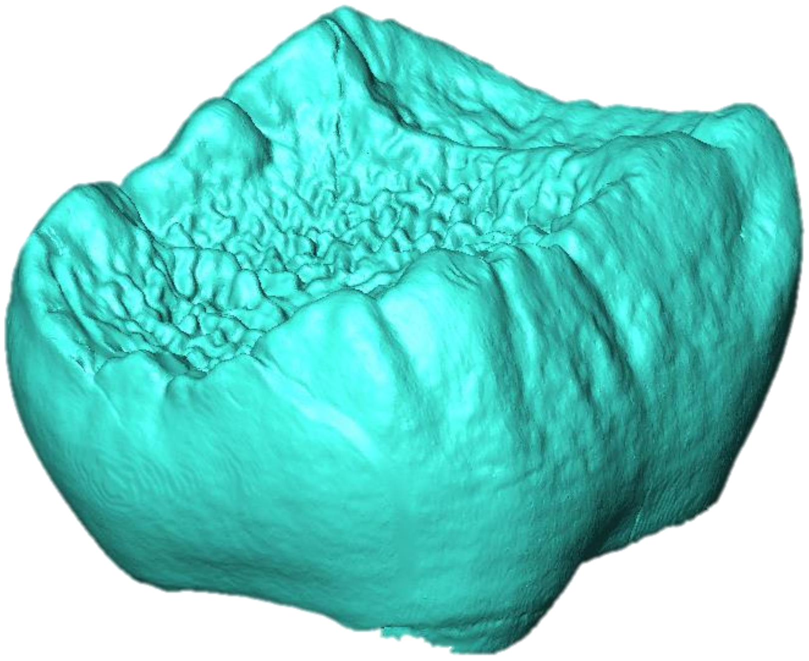

Cacajao calvus – ZMB 18480 LRM2

D)

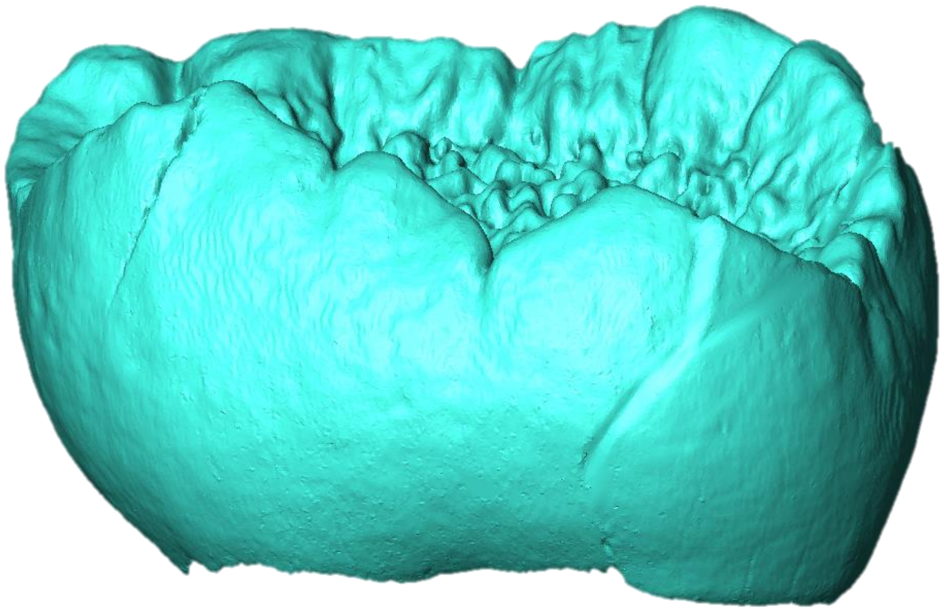

Cacajao calvus – ZMB 18480 LRM2

E)

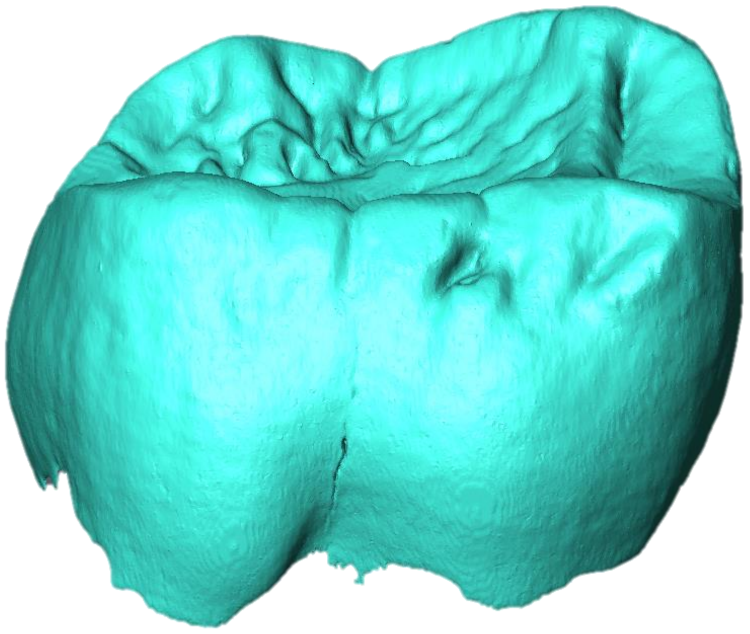

Chiropotes satanas – ZMB 85177 LRM1

F)

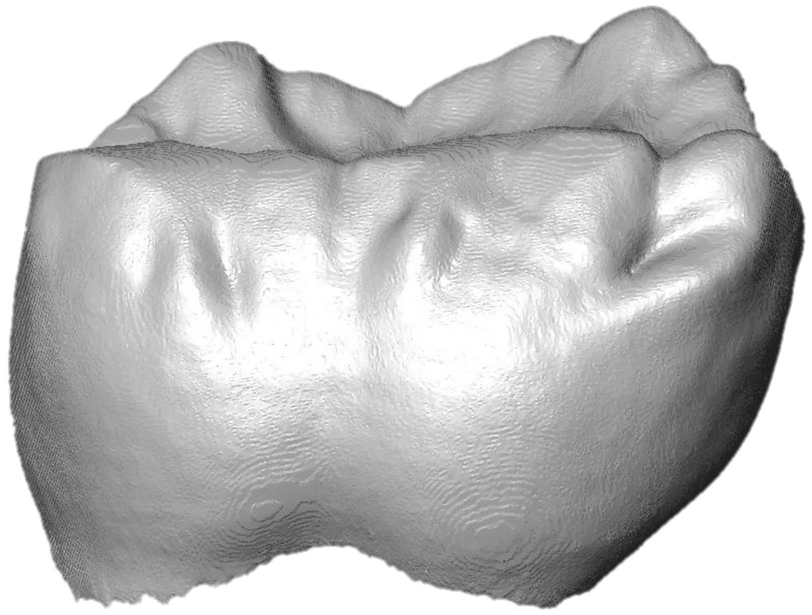

Chiropotes satanas – ZMB 16485 LLM2

C)

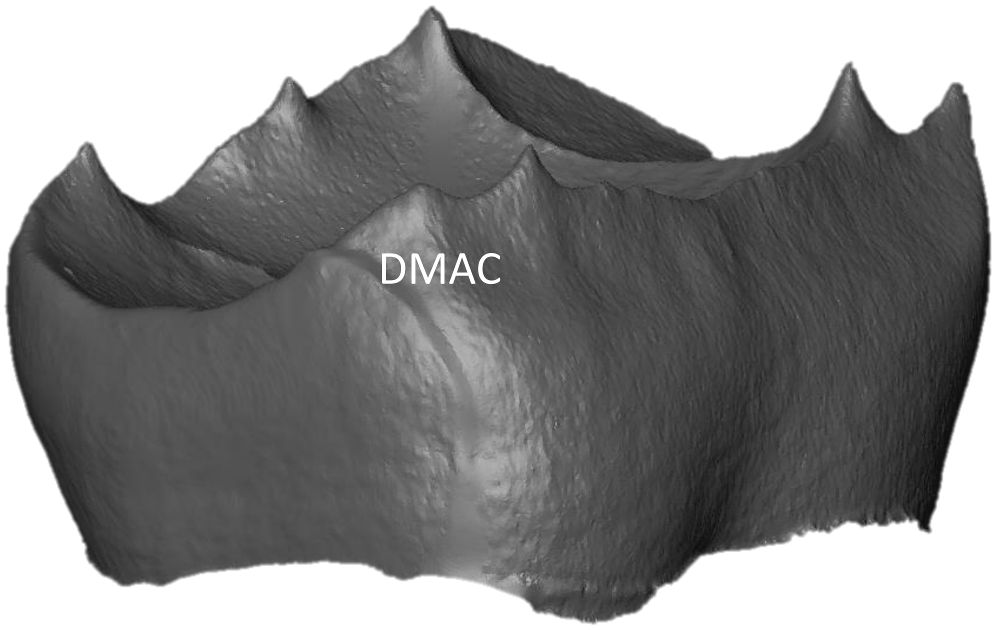

Cacajao calvus – ZMB 18480 LRM2

D)

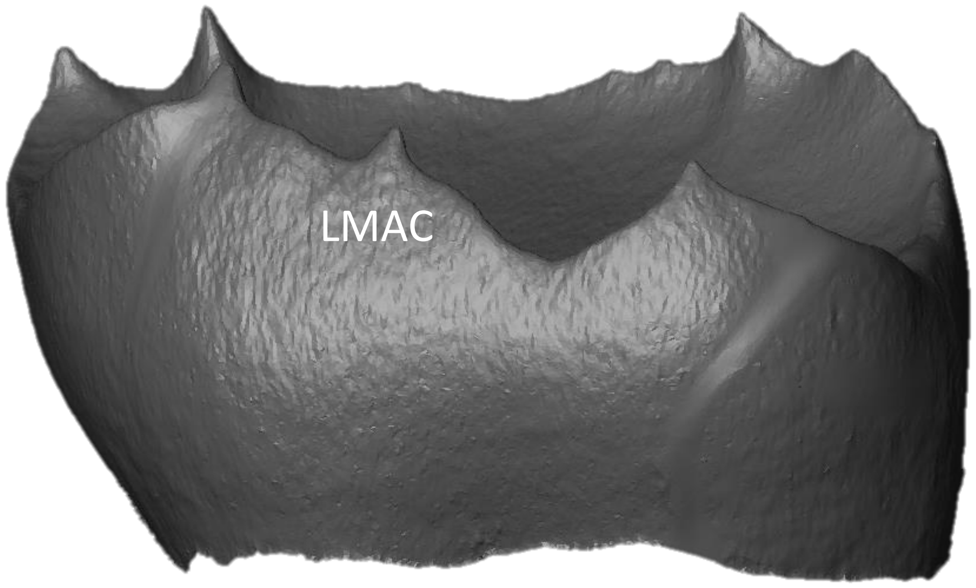

Cacajao calvus – ZMB 18480 LRM2

E)

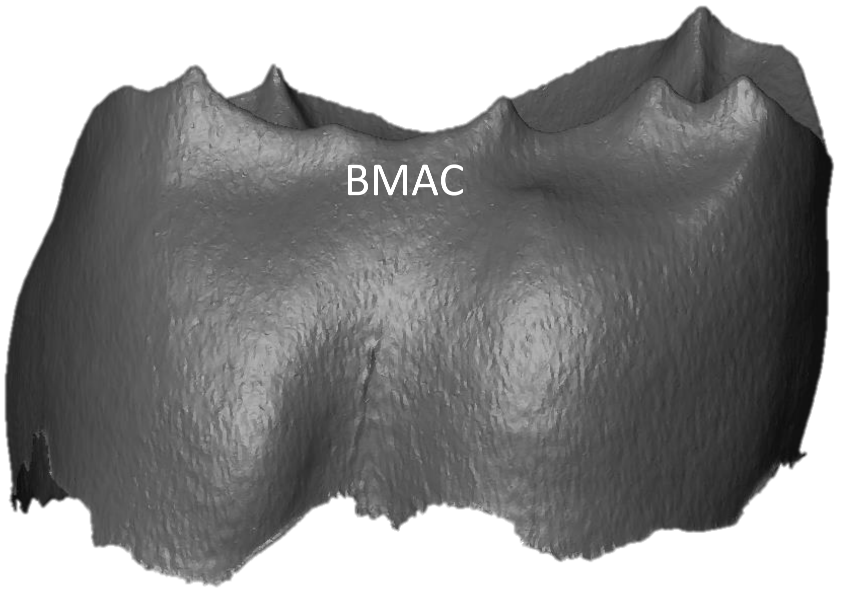

Chiropotes satanas – ZMB 85177 LRM1

F)

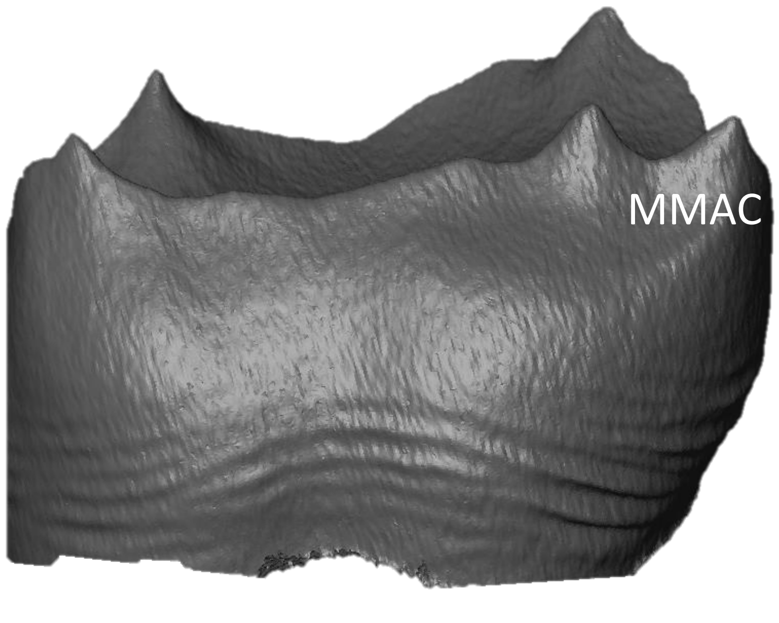

Chiropotes satanas – ZMB 16485 LLM2

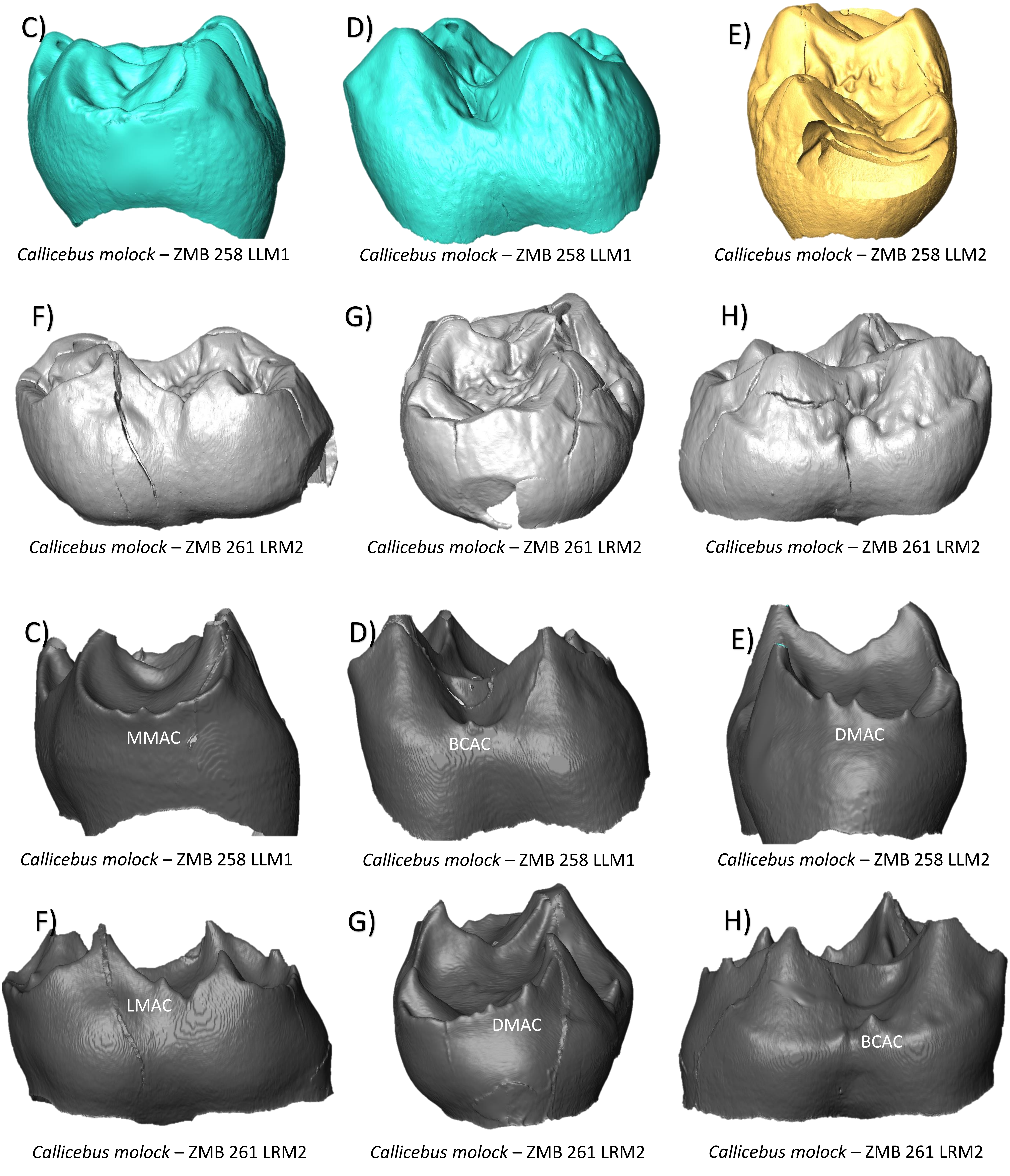
C)
Callicebus molock – ZMB 258 LLM1
D)
Callicebus molock – ZMB 258 LLM1
E)
Callicebus molock – ZMB 258 LLM2
F)
Callicebus molock – ZMB 261 LRM2
G)
Callicebus molock – ZMB 261 LRM2
H)
Callicebus molock – ZMB 261 LRM2
C)
MMAC
Callicebus molock – ZMB 258 LLM1
D)
BCAC
Callicebus molock – ZMB 258 LLM1
E)
DMAC
Callicebus molock – ZMB 258 LLM2
F)
LMAC
Callicebus molock – ZMB 261 LRM2
G)
DMAC
Callicebus molock – ZMB 261 LRM2
H)
BCAC
Callicebus molock – ZMB 261 LRM2

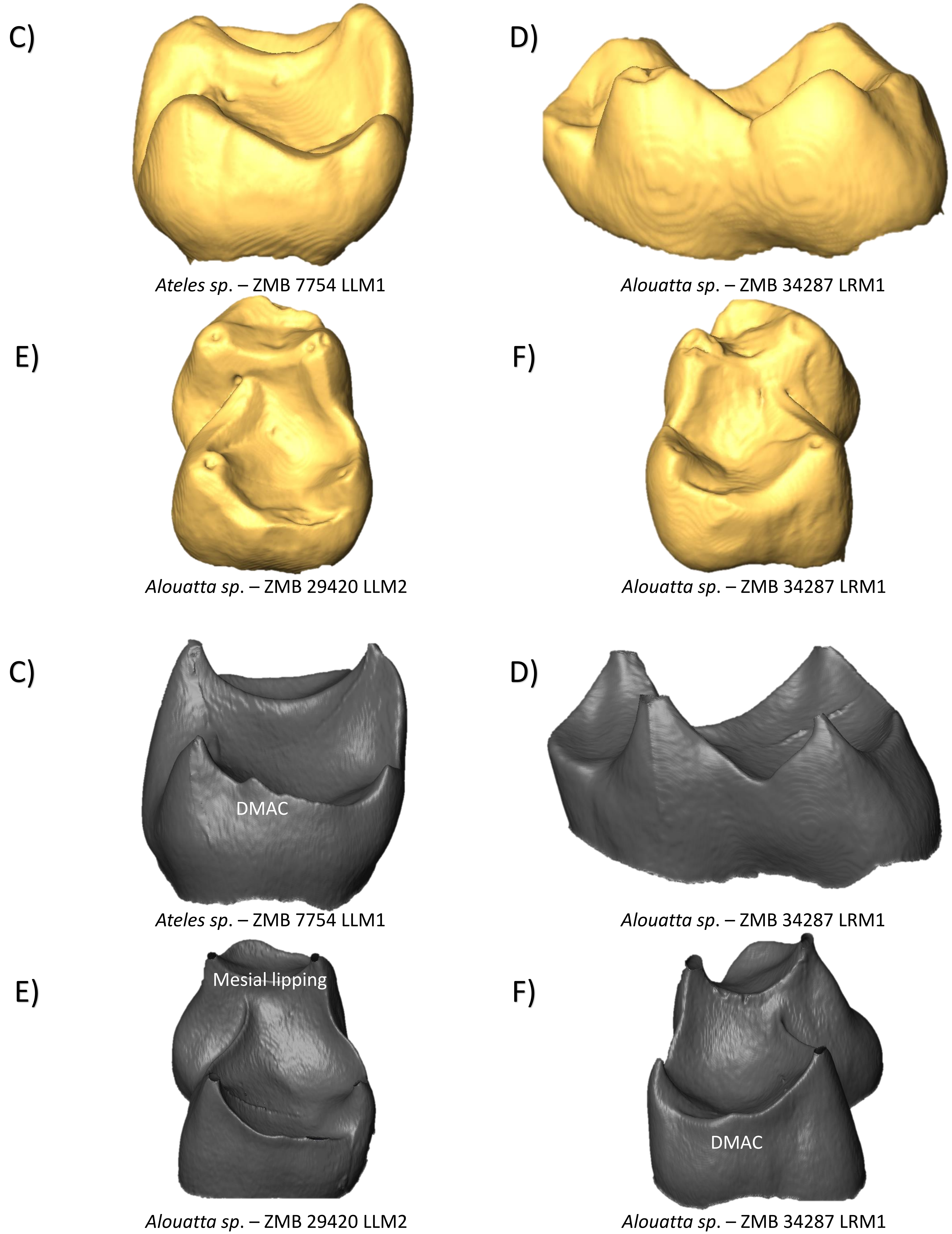
C)
Ateles sp. – ZMB 7754 LLM1
D)
Alouatta sp. – ZMB 34287 LRM1
E)
Alouatta sp. – ZMB 29420 LLM2
F)
Alouatta sp. – ZMB 34287 LRM1
C)
DMAC
Ateles sp. – ZMB 7754 LLM1
D)
Alouatta sp. – ZMB 34287 LRM1
E)
Mesial lipping
Alouatta sp. – ZMB 29420 LLM2
F)
DMAC
Alouatta sp. – ZMB 34287 LRM1

C)

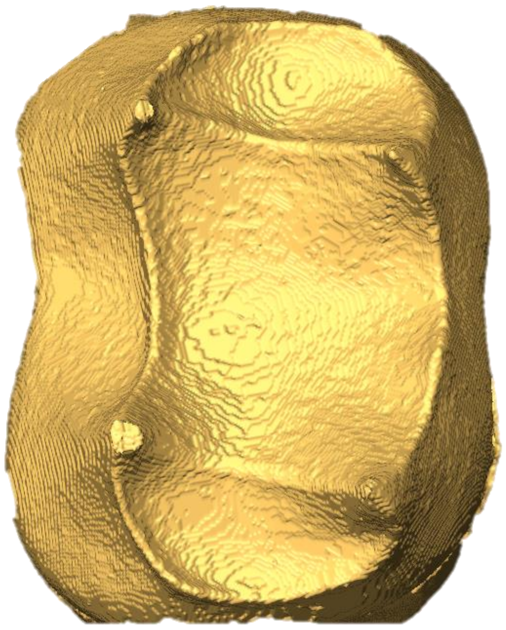

Cercopithecus mitis – ZMB 87536 LLM2

D)

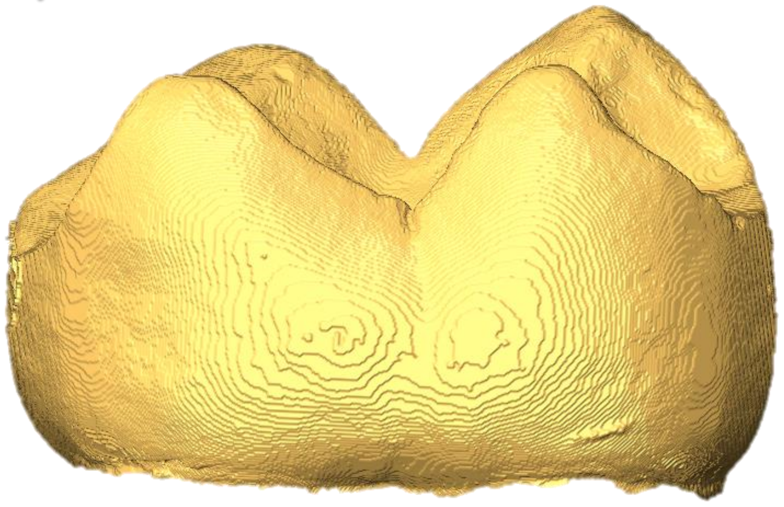

Chlorocebus aethiops - ZMB 87509 LRM2

E)

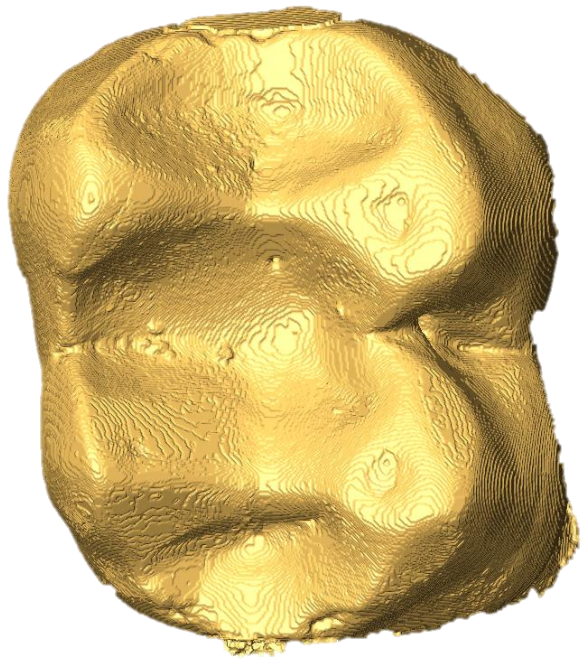

Miopithecus talapoin – ZMB 4943 LRM2

F)

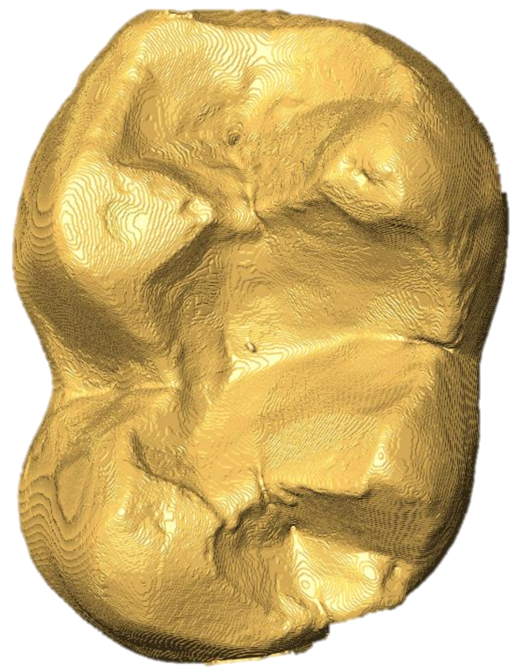

Erythrocebus patas – ZMB 11864 LLM2

C)

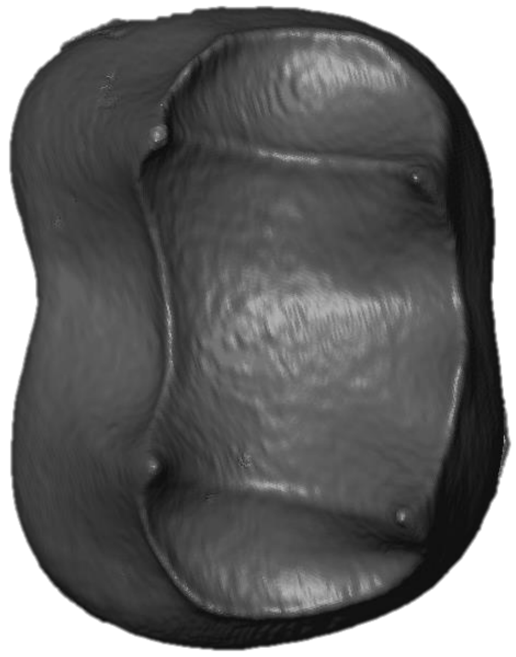

Cercopithecus mitis – ZMB 87536 LLM2

D)

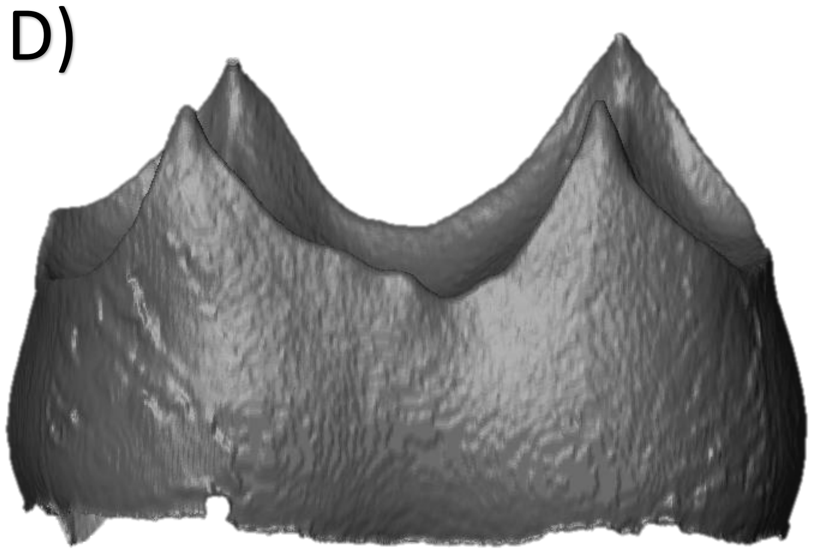

Chlorocebus aethiops - ZMB 87509 LRM2

E)

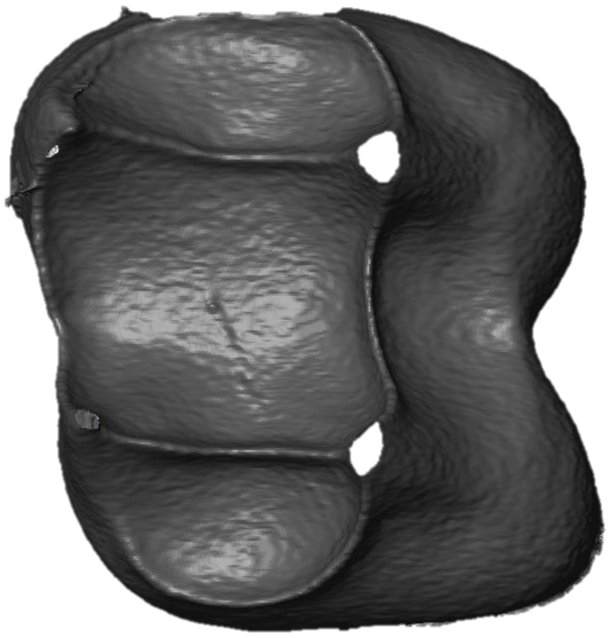

Miopithecus talapoin – ZMB 4943 LRM2

F)

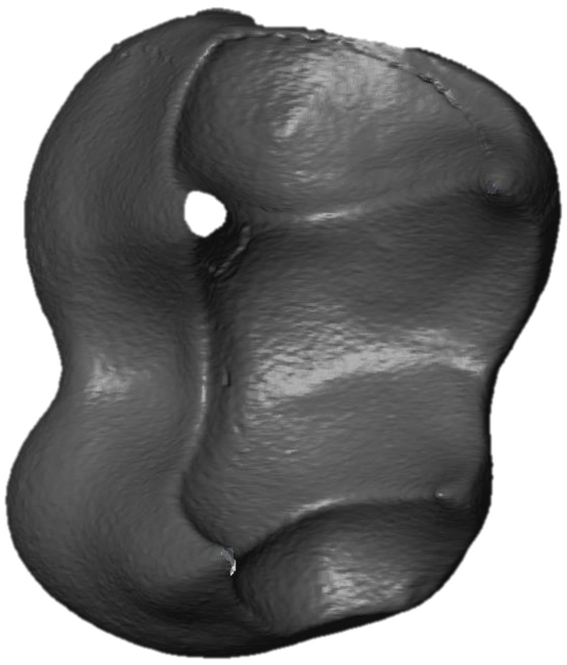

Erythrocebus patas – ZMB 11864 LLM2

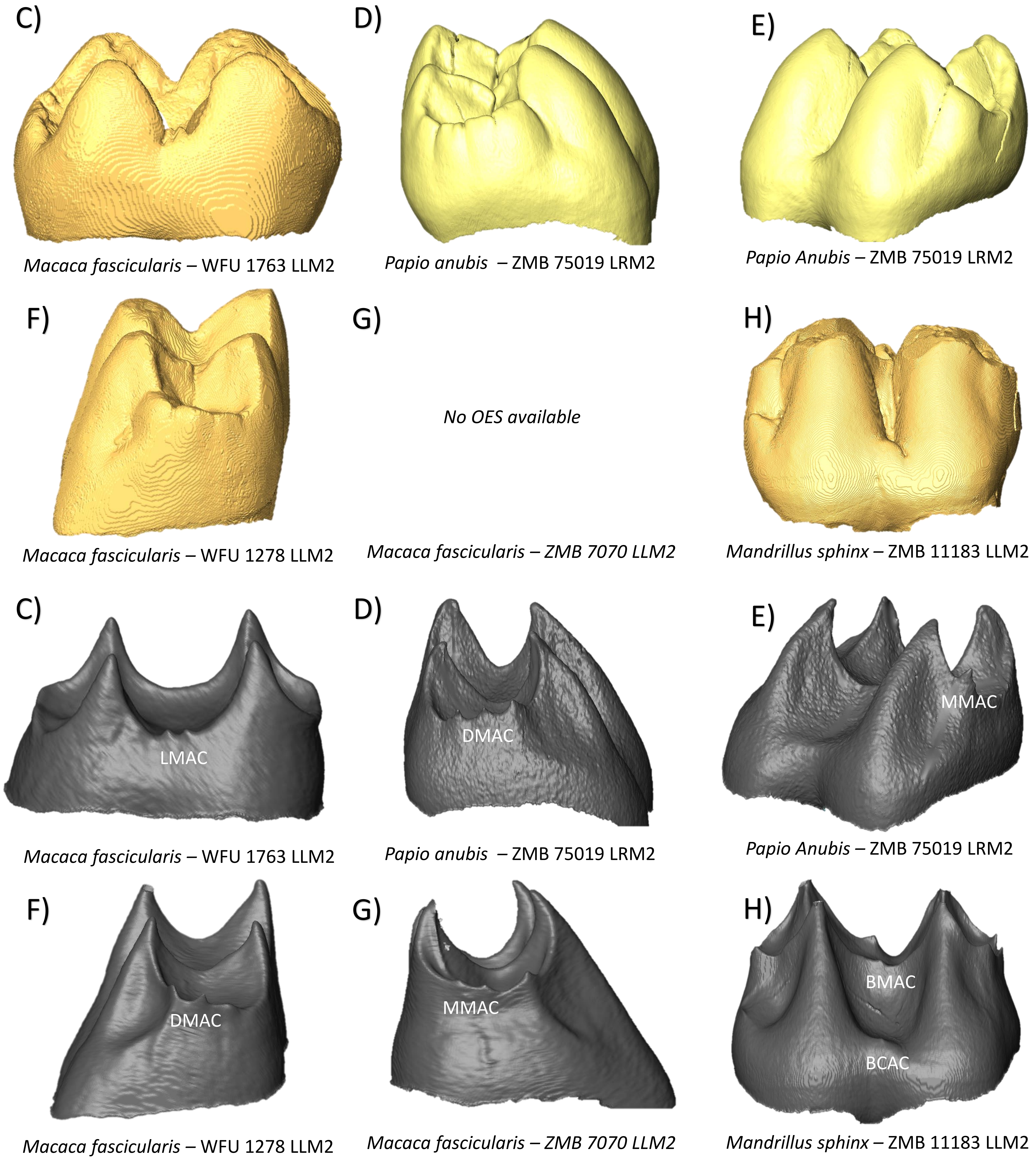
C)
D)
E)
Macaca fascicularis – WFU 1763 LLM2
Papio anubis – ZMB 75019 LRM2
Papio Anubis – ZMB 75019 LRM2
F)
G)
H)
No OES available
Macaca fascicularis – WFU 1278 LLM2
Macaca fascicularis – ZMB 7070 LLM2
Mandrillus sphinx – ZMB 11183 LLM2
C)
D)
E)
LMAC
DMAC
MMAC
Macaca fascicularis – WFU 1763 LLM2
Papio anubis – ZMB 75019 LRM2
Papio Anubis – ZMB 75019 LRM2
F)
G)
H)
DMAC
MMAC
BMAC
BCAC
Macaca fascicularis – WFU 1278 LLM2
Macaca fascicularis – ZMB 7070 LLM2
Mandrillus sphinx – ZMB 11183 LLM2

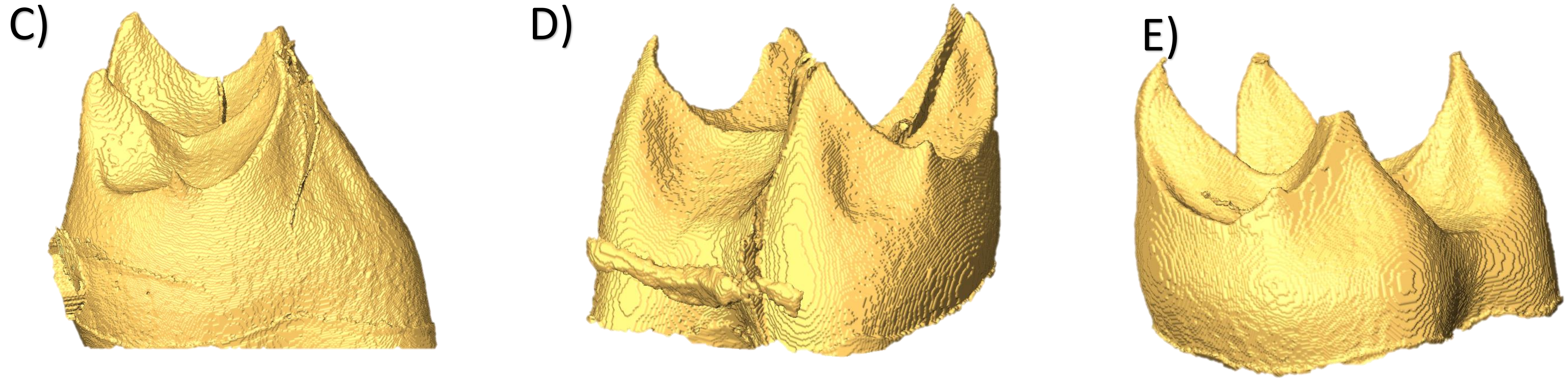

Colobus guereza – ZMB 45404 LRM2 *Presbytis melalophos* – ZMB 73048 LRM2 *Presbytis melalophos* – ZMB 73024 LLM2

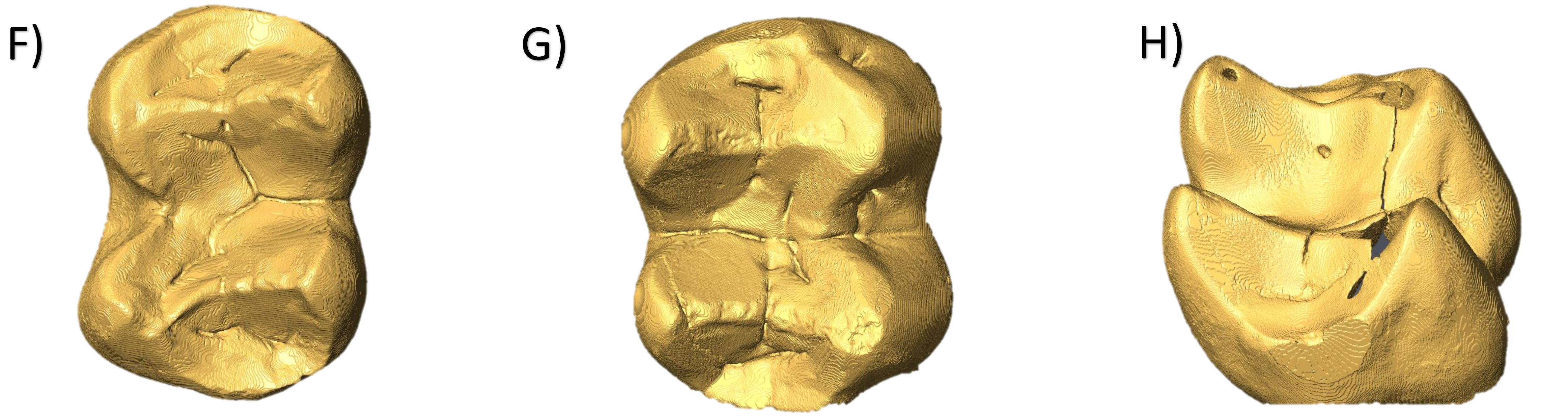

Nasalis larvatus – ZMB 72215 LLM2 *Trachypithecus cristatus* – ZMB 43652 LRM2 *Trachypithecus cristatus* – ZMB 42747 LRM2

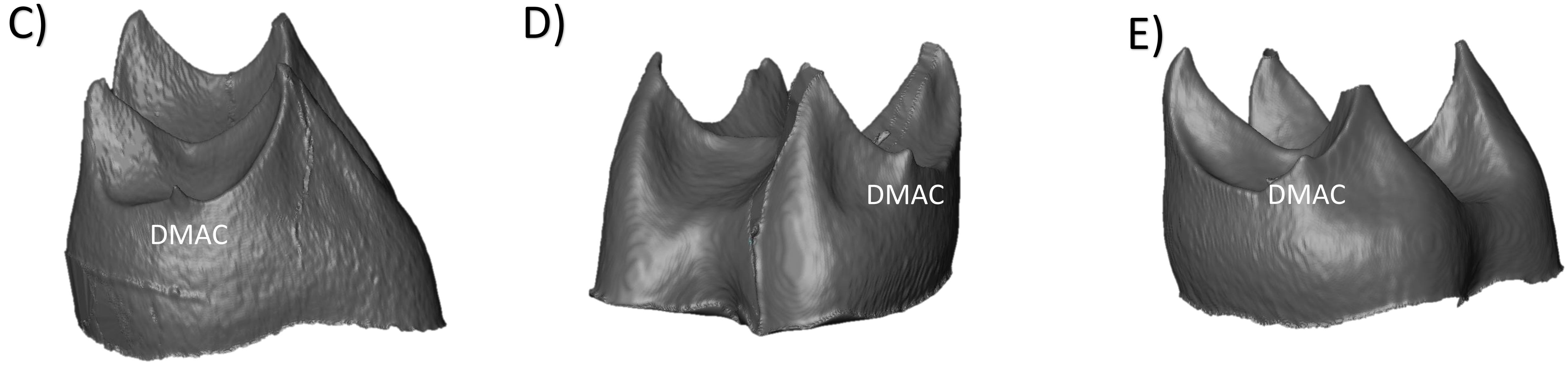

Colobus guereza – ZMB 45404 LRM2 *Presbytis melalophos* – ZMB 73048 LRM2 *Presbytis melalophos* – ZMB 73024 LLM2

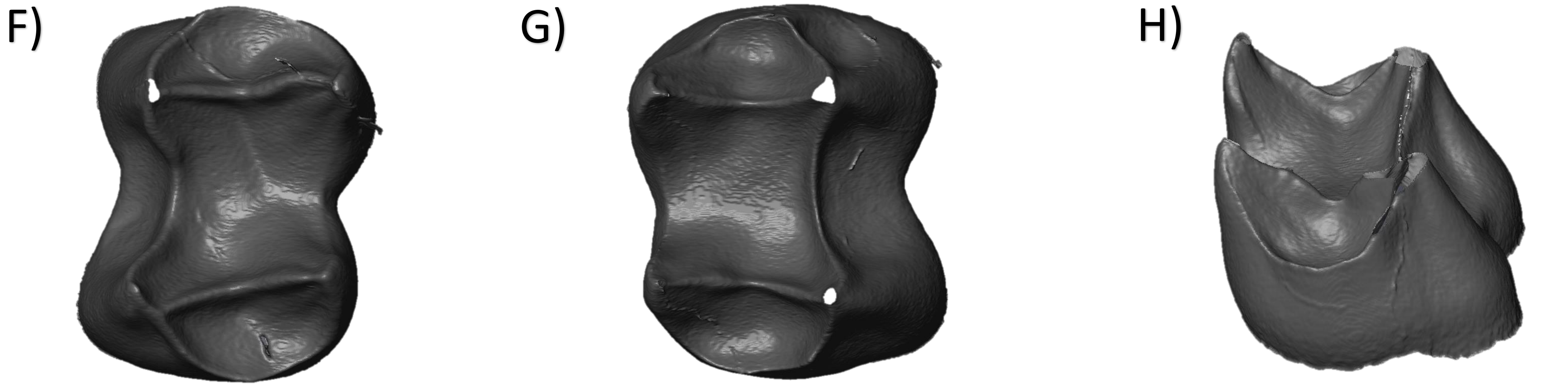

Nasalis larvatus – ZMB 72215 LLM2 *Trachypithecus cristatus* – ZMB 43652 LRM2 *Trachypithecus cristatus* – ZMB 42747 LRM2

C)

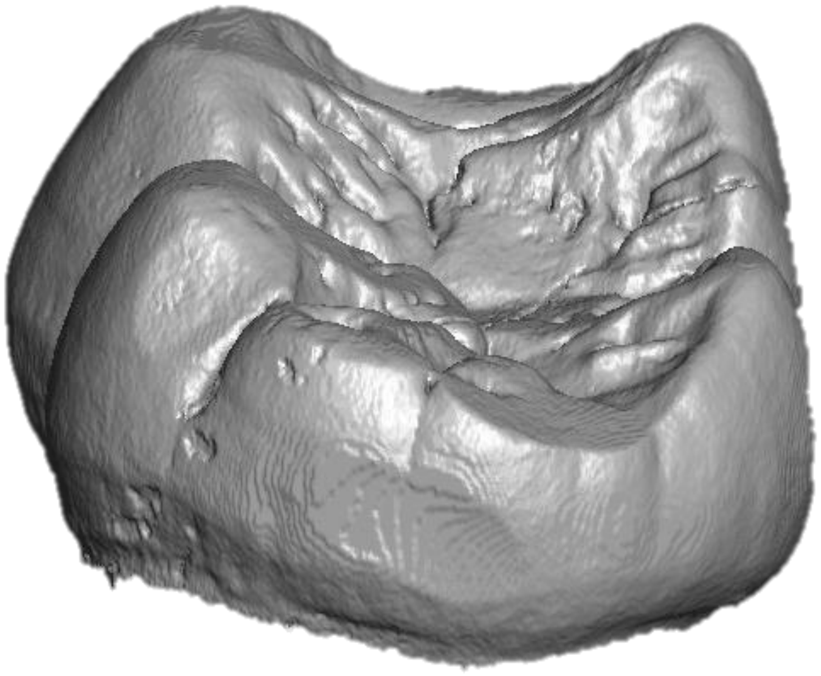

Pan troglodytes verus – MPI-EVA 11800 LLM2

D)

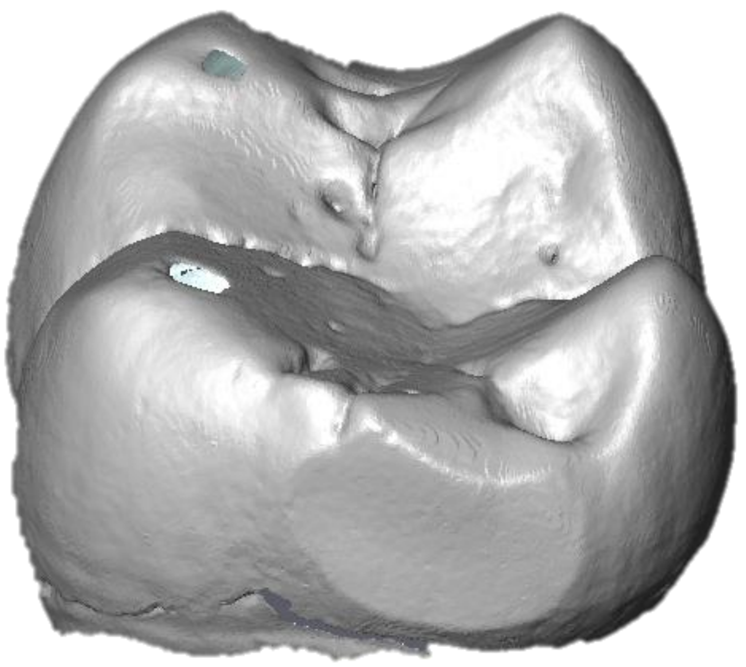

Pan troglodytes verus – MPI-EVA 15008 LLM1

E)

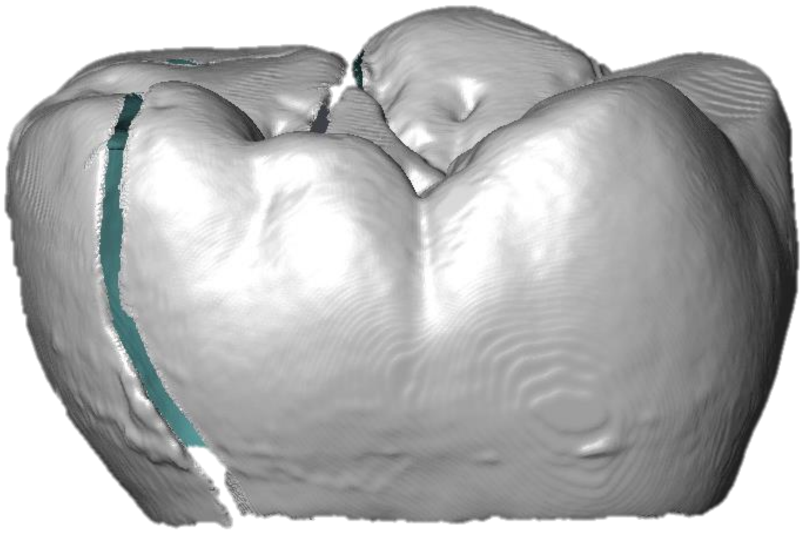

Homo sapiens – R433 LRM1

F)

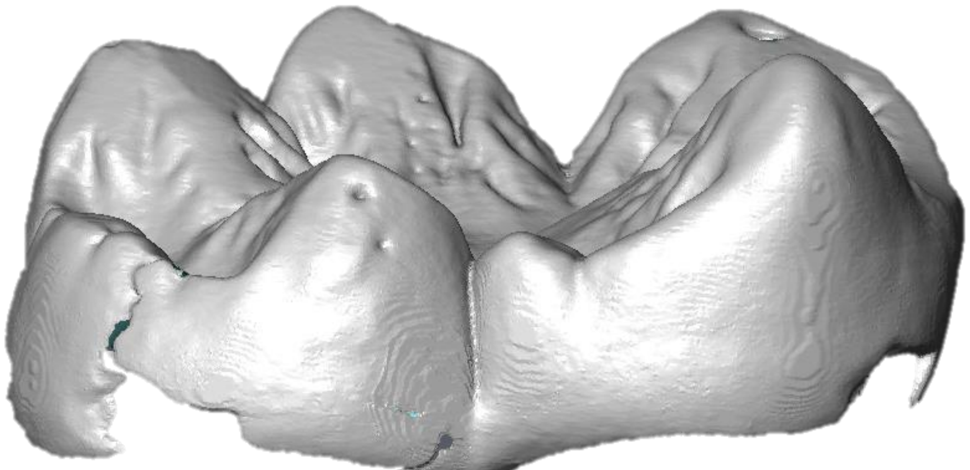

Gorilla gorilla– NMNH 545037 LRM2

C)

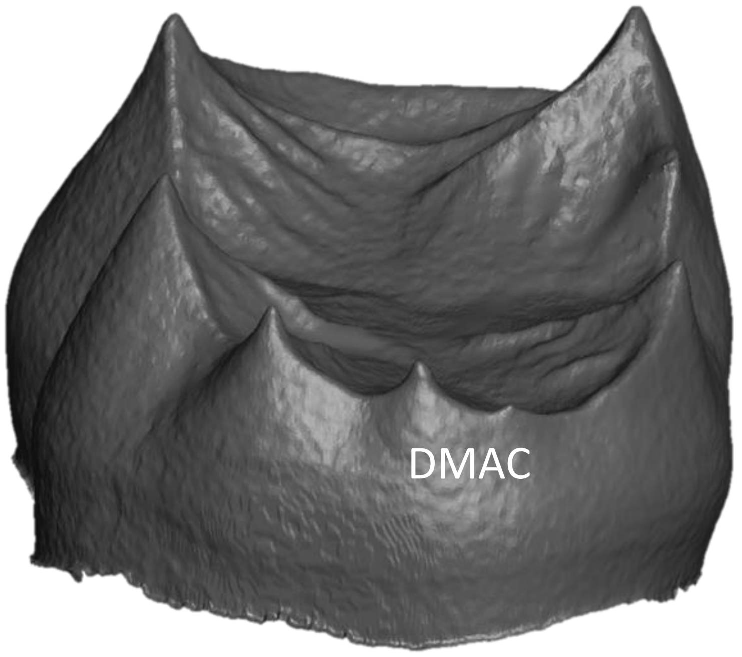

Pan troglodytes verus – MPI-EVA 11800 LLM2

D)

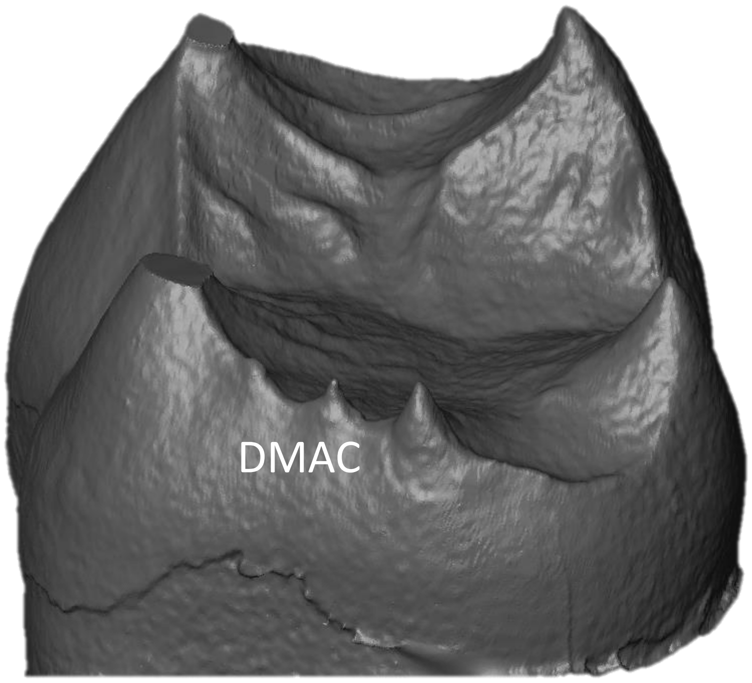

Pan troglodytes verus – MPI-EVA 15008 LLM1

E)

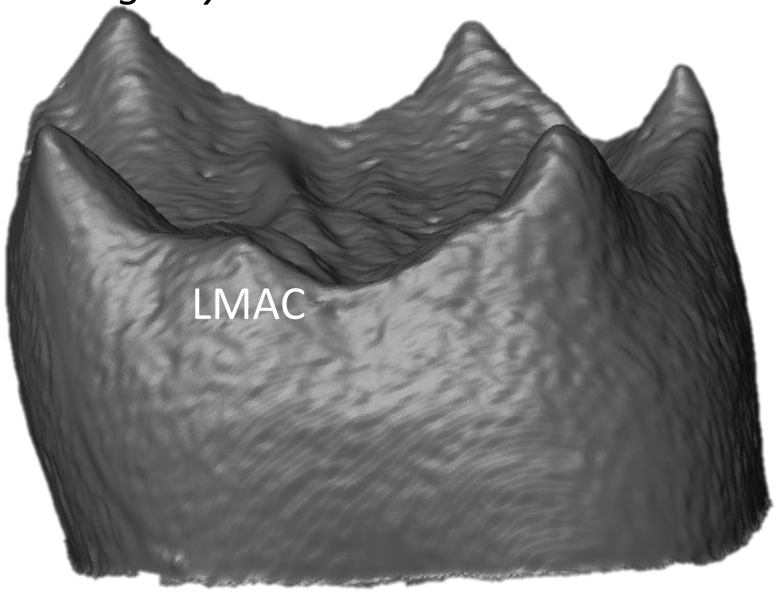

Homo sapiens – R433 LRM1

F)

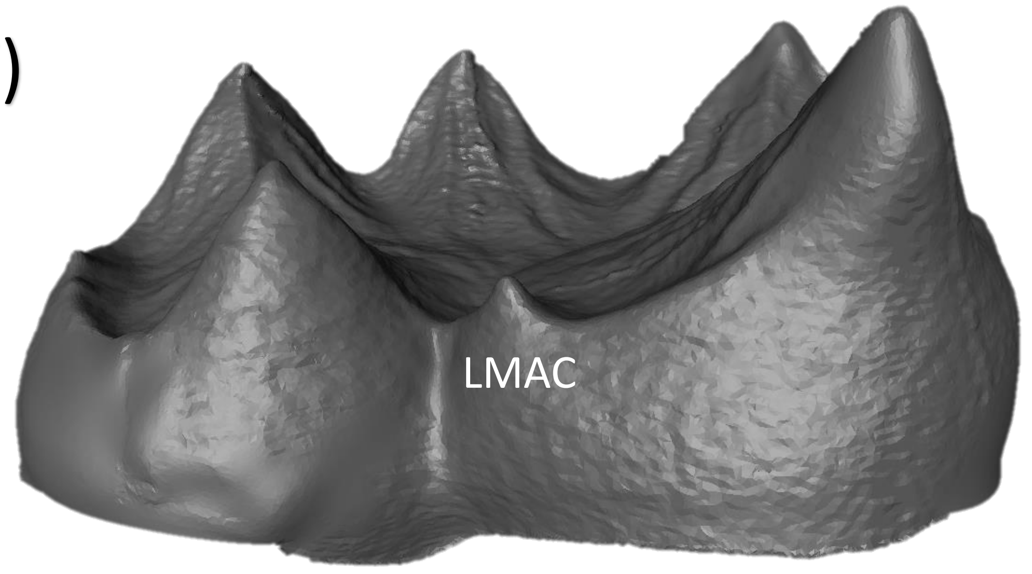

Gorilla gorilla– NMNH 545037 LRM2

C)

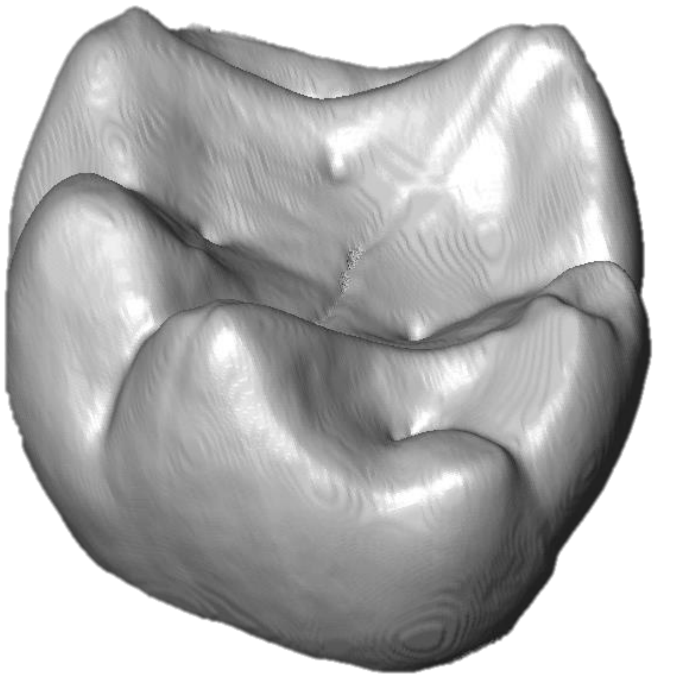

Hylobates muelleri– AMNH 103726 LLM2

D)

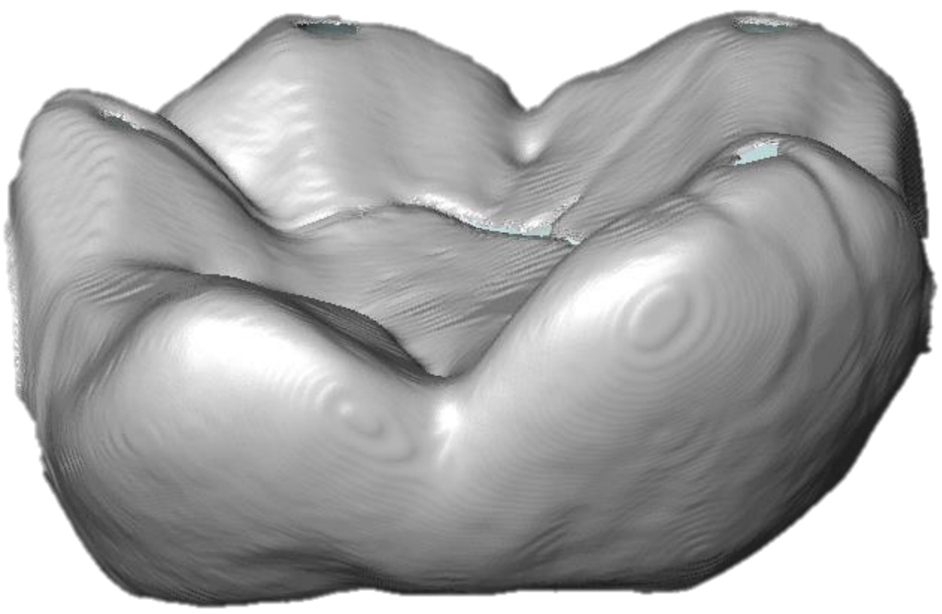

Hylobates muelleri– AMNH 103726 LLM1

E)

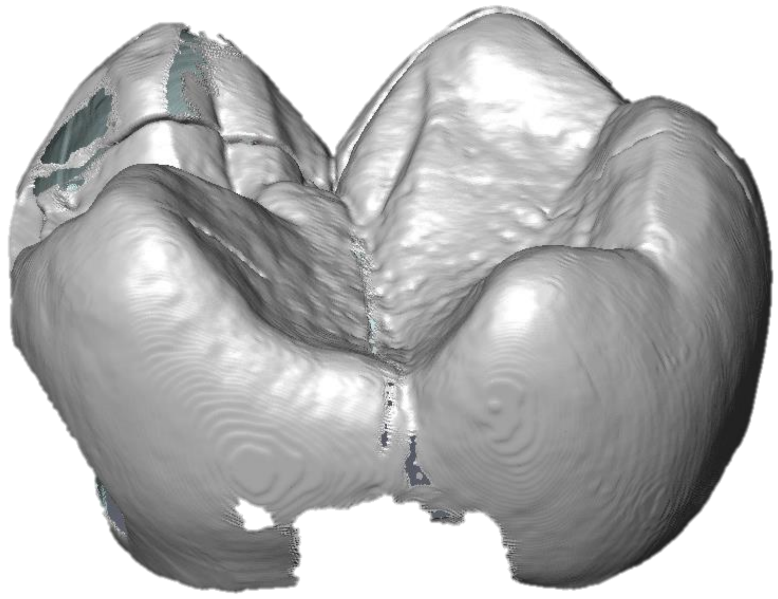

Symphalangus syndactylus– AMNH 100048 LRM1

F)

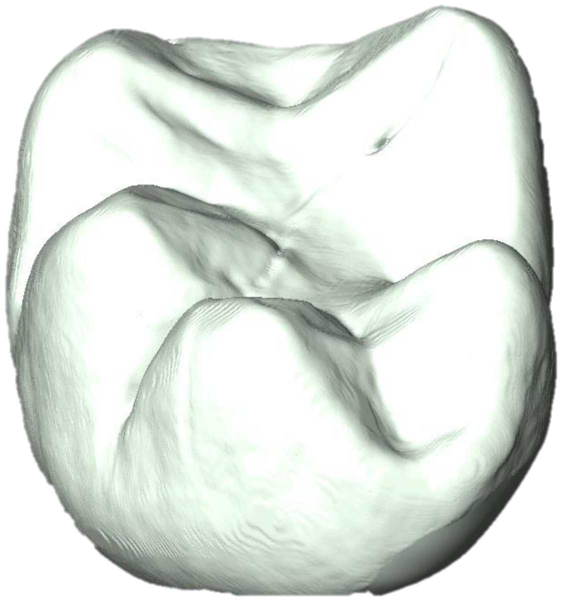

Symphalangus syndactylus– AMNH 102193 LLM2

C)

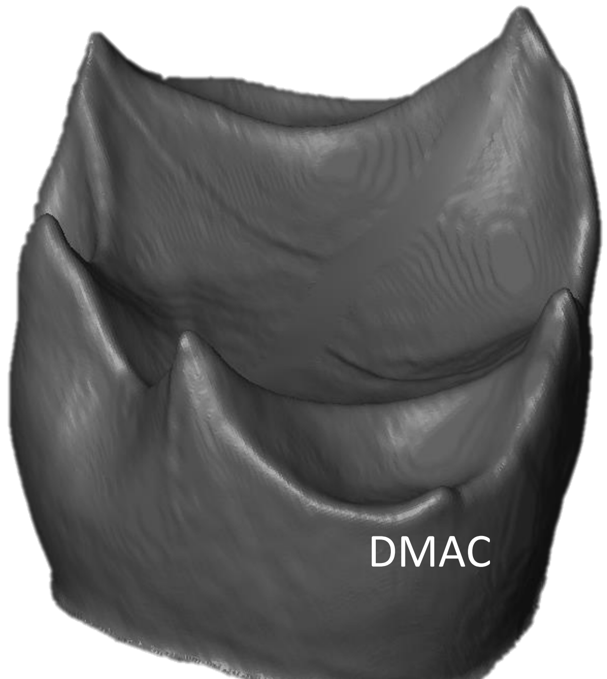

Hylobates muelleri– AMNH 103726 LLM2

D)

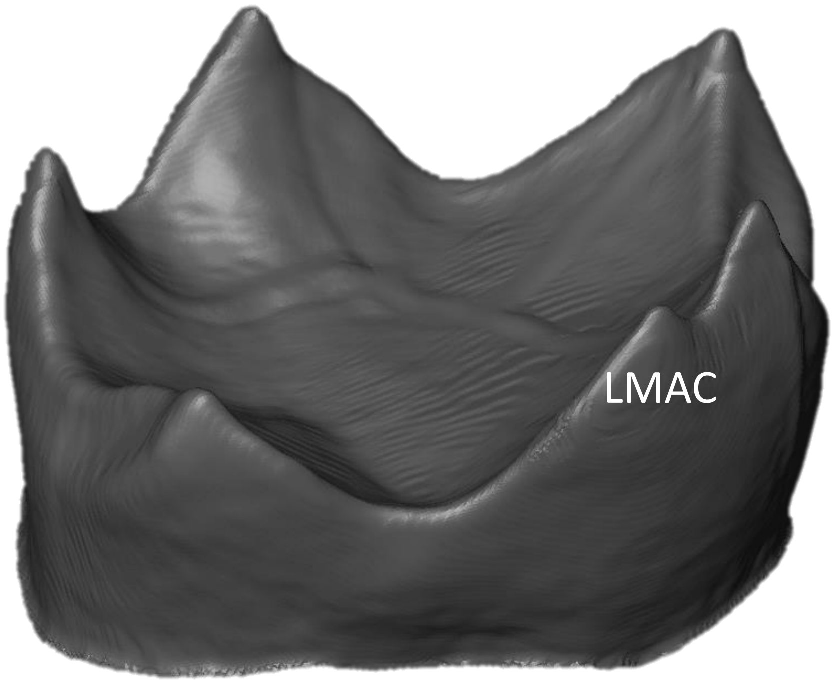

Hylobates muelleri– AMNH 103726 LLM1

E)

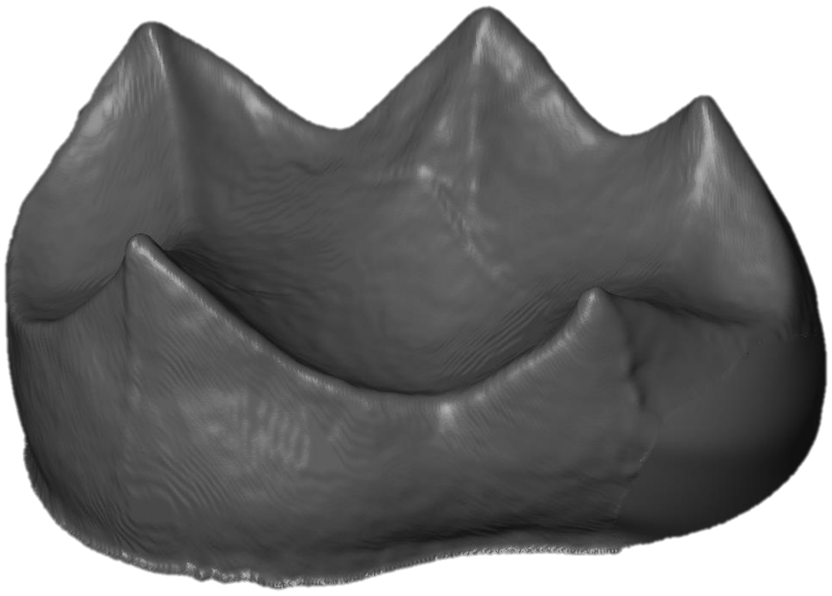

Symphalangus syndactylus– AMNH 100048 LRM1

F)

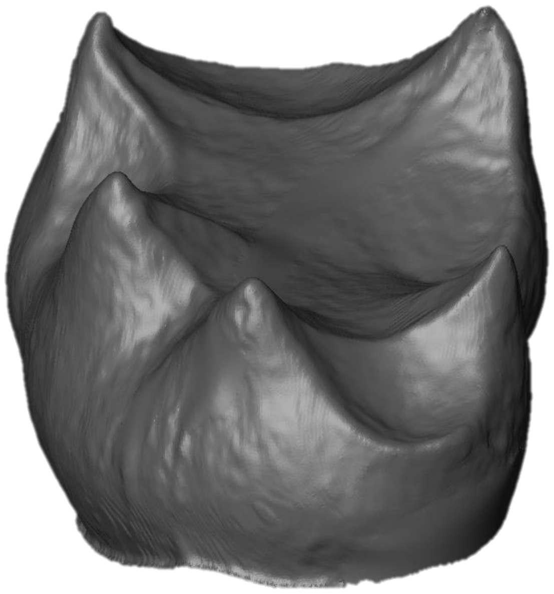

Symphalangus syndactylus– AMNH 102193 LLM2
