# Supplementary material for: Primate tooth crown nomenclature revisited
Source: PeerJ. 2023 Jan 12;11:e14523. doi: 10.7717/peerj.14523 (PMC9840859; doi:10.7717/peerj.14523)
Supplement: File S3 [file peerj-11-14523-s003.docx]

| **Clade** | **Current Variation** | **New Variation** |
| --- | --- | --- |
| **Lemuridae** | Four primary cusps, hypoconulid, and T. intermedium. | Four primary cusps, plus DMAC, LMAC, BMAC and BCAC expression. |
|  |  |  |
| **Lepilemuridae** | Four primary cusps (entoconid may be metastylid), and mesostylid. | Four primary cusps, plus MMAC and DMAC expression. |
|  |  |  |
| **Cheirogaleidae** | Four primary cusps. | Four primary cusps, plus BMAC expression. |
|  |  |  |
| **Indriidae** | Four primary cusps, parastylid, and T. intermedium. | Four primary cusps, plus MMAC and LMAC expression. |
|  |  |  |
| **Galagidae** | Four primary cusps, protostylid, and unnamed conulid on cristid obliqua. | Four primary cusps, plus MMAC, DMAC, BMAC, and BCAC expression |
|  |  |  |
| **Lorisidae** | Four primary cusps, and parastylid. | Four primary cusps plus MMAC and DMAC expression. |
|  |  |  |
| **Tarsioidea** | Five primary cusps. | Five primary cusps, plus LMAC, DMAC, BMAC, and BCAC expression. |
|  |  |  |
| **Callitrichinae** | Four primary cusps. | Four primary cusps, plus LMAC and BCAC expression. |
|  |  |  |
| **Cebinae** | Four primary cusps. | Four primary cusps, plus MMAC, DMAC, and BCAC expression. |
|  |  |  |
| **Pitheciinae** | Four primary cusps. | Four primary cusps, plus MMAC, LMAC, DMAC, and BMAC expression. |
|  |  |  |
| **Callicebinae** | Four primary cusps, and distostylid. | Four primary cusps, plus MMAC, DMAC, and BCAC expression. |
|  |  |  |
| **Atelinae** | Four primary cusps, hypoconulid, and paraconid. | Four primary cusps, plus DMAC expression. |
|  |  |  |
| **Cercopithecini** | Four primary cusps, and protostylid. | Four primary cusps, plus BCAC expression. |
|  |  |  |
| **Papionini** | Four primary cusps, ectostylid, and T. intermedium. | Four primary cusps, plus MMAC, LMAC, DMAC, BMAC, and BCAC expression. |
|  |  |  |
| **Colobinae** | Four primary cusps, and T. intermedium. | Four primary cusps, plus LMAC and DMAC expression. |
|  |  |  |
| **Hominidae** | Four primary cusps, hypoconulid, c6, and c7. | Four primary cusps, hypoconulid, LMAC, and DMAC expression |
|  |  |  |
| **Hylobatidae** | Four primary cusps, hypoconulid, and T. intermedium. | Four primary cusps, hypoconulid, LMAC, and DMAC expression. |
